# Supplementary material for: Nanosecond-latency all-optical fiber sensing with in-sensor computing
Source: Light Sci Appl. 2026 May 25;15:251. doi: 10.1038/s41377-026-02265-x (PMC13201864; doi:10.1038/s41377-026-02265-x)
Supplement: Supplementary file 1 — Supplemental Materials [file 41377_2026_2265_MOESM1_ESM.pdf]

Supplementary information for

Nanosecond-latency all-optical fiber sensing with in-sensor computing

Yu Tao[1], Yangyang Wan[1,*], Ziwen Long[1], Wenjia Zhang[1], Jiangbing Du[1], Zuyuan He[1]

[1]State Key Laboratory of Photonics and Communications, Shanghai Jiao Tong University, Shanghai 200240, China.

[*]Corresponding author: YangyangWan@sjtu.edu.cn

Contents

Supplementary Note 1. Theoretical modeling and analysis of MMFs **2**

Supplementary Note 2. Correlation between speckles and MMF memory effect **3**

Supplementary Note 3. Fiber responses to distinct measurands **4**

Supplementary Note 4. Cross-sensitivity analysis and multi-parameter sensing **6**

Supplementary Note 5. Forward propagation model based on optical field diffraction **7**

Supplementary Note 6. Discussion on training methods **8**

Supplementary Note 7. Genetic algorithm for AOFS-IC training **8**

Supplementary Note 8. Implementation process of AOFS-IC **10**

Supplementary Note 9. Computational paradigm of AOFS-IC **11**

Supplementary Note 10. Computational performance of AOFS-IC **12**

Supplementary Note 11. Sensing performance of AOFS-IC for various other measurands **14**

Supplementary Note 12. Comparison with classical fiber-optic sensing systems **16**

Supplementary Tables **19**

Supplementary Figures **21**

Supplementary Videos **37**

Supplementary References **38**

Supplementary Note 1. Theoretical modeling and analysis of MMFs

Multimode fibers (MMFs) are optical waveguides characterized by core diameters significantly larger than those of single-mode fibers, enabling the propagation of a large number of spatially distinct guided modes. This high modal capacity introduces substantial complexity to the theoretical analysis of light propagation within the fiber, particularly in the modeling of mode structures, intermodal coupling, and output field reconstruction.

A foundational theoretical framework[1] for describing the guided modes in MMFs is the concept of propagation-invariant modes (PIMs). For a step-index fiber with core radius a, the electric field distribution of a single mode in cylindrical coordinates (r, ϕ) can be expressed as

$$E(r,\phi) = \begin{cases} A_l J_l(ur)e^{il\phi}, & r < a \quad \text{(core region)} \\ B_l K_l(wr)e^{il\phi}, & r \geq a \quad \text{(cladding region)} \end{cases} \tag{S1}$$

where J_l and K_l denote the Bessel function of the first kind and the modified Bessel function of the second kind, respectively, both of order l. The parameters $u = \sqrt{k_0^2 n_{\text{core}}^2 - \beta^2}$ and $w = \sqrt{\beta^2 - k_0^2 n_{\text{clad}}^2}$ are the transverse wavenumbers in the core and cladding regions, respectively, with β representing the longitudinal propagation constant. The integer l corresponds to the azimuthal mode order (or angular momentum quantum number), and ϕ is the azimuthal angle. The complex amplitudes A_l and B_l are determined from the boundary conditions enforcing field continuity at the core-cladding interface.

At the output facet of an MMF, the interference of the various supported modes gives rise to complex spatial patterns, commonly observed as speckle. These output intensity patterns are governed by the coherent superposition of the excited modal fields and can be written as

$$I(r,\phi) = \left| \sum_{l,m} c_{lm} E_{lm}(r,\phi) \right|^2 \tag{S2}$$

where c_{lm} denotes the complex excitation coefficient for the mode indexed by (l, m), incorporating both amplitude and phase information. The spatial structure of the output field is thus highly sensitive to the modal excitation profile at the input, as well as to propagation effects such as modal dispersion and intermodal mixing.

To model the transformation of light through the MMF in the modal domain, we adopt a transmission matrix formalism that acts on the mode coefficient space. Specifically, we express the output modal coefficients $\boldsymbol{c}_{\text{out}}$ as a linear transformation of the input modal coefficients $\boldsymbol{c}_{\text{in}}$ via $\boldsymbol{c}_{\text{out}} = \mathrm{T}_{\text{mode}} \cdot \boldsymbol{c}_{\text{in}}$, where T_{mode} is a mode transmission matrix that encapsulates intermodal coupling, propagation-induced phase accumulation, and possible perturbations due to fiber imperfections or external influences. Each element T_{ij} of the matrix quantifies the coupling from input mode j to output mode i, and is typically written as $T_{ij} = \sqrt{v_{ij}}\, e^{i\phi_{ij}}$, with v_{ij} denoting the power transfer efficiency and ϕ_{ij} the associated phase shift between modes. This provides a compact and powerful framework for analyzing modal transformations and enables wavefront control and optical information processing through MMFs.

Based on the above classical theory, we conduct numerical simulations to explore the modal properties of a step-index MMF under realistic parameter settings (Fig. S1). The operating wavelength is set to $\lambda = 1.55$ μm in air. The fiber is assumed to have a core diameter of $\mathrm{D} = 105$ μm and

a numerical aperture of $\mathrm{NA} = 0.22$. The core refractive index is specified as $n_{\text{core}} = 1.46$, and the corresponding cladding index is computed from the NA relation as $n_{\text{clad}} = \sqrt{n_{\text{core}}^2 - \mathrm{NA}^2} = 1.44$. Under these conditions, the calculated mode spectrum revealed that the fiber supports more than 1100 guided modes.

The high mode count of MMFs not only provides substantial information-carrying capacity but also enables the potential for nonlinear information encoding. MMFs effectively function as nonlinear and scattering media-not due to intrinsic material nonlinearity, but as a consequence of their high modal dimensionality and complex internal propagation dynamics. Even a spatially simple input can excite hundreds or thousands of guided modes in a large-core MMF. As light propagates, each mode accrues a unique phase owing to its distinct propagation constant. The resulting coherent superposition gives rise to highly structured interference patterns that are extremely sensitive to the input configuration. In addition, factors such as modal dispersion, fabrication imperfections, and nonlinear effects at high optical powers further enhance the complexity of light propagation within MMFs. These combined influences render the input-output relationship effectively chaotic. As such, MMFs are better understood as structured yet disordered systems and can be modeled as high-dimensional nonlinear systems. This fundamental property enables the implementation of proposed all-optical fiber sensing architectures across various sensing systems.

Supplementary Note 2. Correlation between speckles and MMF memory effect

To quantitatively characterize the dynamic evolution of mode field distributions in MMFs, we introduce the normalized Pearson cross-correlation (NPCC) coefficient. This metric is defined as:

$$\mathrm{NPCC}(I_1, I_2) = \frac{\sum_{i=1}^{N} \left(I_1(i) - \bar{I}_1\right)\left(I_2(i) - \bar{I}_2\right)}{\sqrt{\sum_{i=1}^{N} \left(I_1(i) - \bar{I}_1\right)^2}\sqrt{\sum_{i=1}^{N} \left(I_2(i) - \bar{I}_2\right)^2}} \tag{S3}$$

Here, $I_1(i)$ and $I_2(i)$ denote the intensity values at the i-th pixel of two speckle patterns, and $\bar{I}_1$, $\bar{I}_2$ are their respective mean intensities. The summation is performed over all N pixels in the region of interest.

The NPCC coefficient ranges from -1 to 1, with values approaching 1 indicating perfect structural correlation and 0 representing complete decorrelation. Crucially, this metric is insensitive to absolute intensity variations while effectively capturing structural similarities between speckle patterns. This property makes it particularly suitable for analyzing coherence-induced speckle features in MMFs. In practice, external perturbations induce progressive decorrelation of MMF-generated speckle patterns due to the memory effect[2] in MMFs. The NPCC provides a sensitive measure of this decorrelation process, enabling quantitative assessment of environmental disturbances on the MMF transmission characteristics.

When the correlation coefficient between two speckle patterns remains high across different states, it can be assumed that transitions between these states induce only minor, approximately linear evolution of the speckle pattern. This property allows us to establish accurate full-range sensing calibration through characterization of speckle patterns at a limited number of discrete measurement states. However, the decorrelation rate fundamentally constrains the sensing performance: excessively rapid decorrelation prevents establishment of continuous mappings between measurement states, while overly slow decorrelation results in speckle variations too subtle for

reliable detection. These competing factors ultimately determine the fundamental limits of sensing resolution and range. Through computational modeling and experimental verification, we systematically investigate the decorrelation characteristics of each measurand and statistically analyze their measurement performance.

Supplementary Note 3. Fiber responses to distinct measurands

Here, we systematically investigates the responses of several optical fibers to various physical measurands, including temperature, axial strain, radial deformation, torsion, bending, and wavelength. Each measurand alters the optical field in a distinct manner, which in turn alters the generated speckle pattern under the promotion of scattering medium (i.e., MMF). Both numerical simulations and experimental validations are presented, providing theoretical support for the deployment of the all-optical fiber sensing architecture with in-sensor computing (AOFS-IC).

Temperature response. The temperature response of optical fibers arises from the thermo-optic effect and thermal expansion. For fiber Bragg gratings (FBGs), the Bragg wavelength is given by $\lambda_B = 2n_{\text{eff}}\Lambda$, where both n_{eff} and Λ depend on temperature. The wavelength shift with temperature can be expressed as:

$$\frac{d\lambda_B}{dT} = \lambda_B(\frac{1}{n}\frac{dn}{dT} + \frac{1}{\Lambda}\frac{d\Lambda}{dT}) \tag{S4}$$

In silica fibers, this coefficient corresponds to a typical sensitivity of approximately 12 pm/°C. In single-mode fibers (SMFs), temperature changes affect the optical path length nL, inducing a phase shift given by:

$$\Delta\phi = \frac{2\pi}{\lambda}\left(L\frac{dn}{dT} + n\frac{dL}{dT}\right)\Delta T \tag{S5}$$

Single phase changes are difficult to detect, but MMFs respond to temperature in a more complex manner. Each mode acquires a temperature-dependent phase $\beta_m(T)L(T)$, while the mode coupling coefficients $c_m(T)$ also vary with temperature. According to Eq. (S2), the output intensity evolves as:

$$I_{\text{out}}(T) = \left|\sum_m c_m(T)\, e^{i\beta_m(T)L(T)} E_m\right|^2 \tag{S6}$$

Consequently, speckle patterns change nonlinearly and sensitively with temperature. However, the challenges in achieving precise and iterative temperature control have prevented experimental validation of proposed AOFS-IC for temperature measurement. We only provide examples of temperature decorrelation of MMF speckle in simulation, as shown in Fig. S4a.

Axial strain response. Axial strain significantly affects both FBGs and MMFs through different mechanisms. The response of FBG to strain can actually be approximated by the response to wavelength shift (∼1.2 pm/με), with the difference being that the FBG reflection spectrum contains a wider frequency component, which reduces the speckle decorrelation speed caused by wavelength changes. The response of MMFs to strain is directly reflected in the increase of optical path length, which can be expressed as:

$$I_{\text{out}}(\epsilon) = \left|\sum_m c_m\, e^{i\beta_m L(1+\epsilon)} E_m\right|^2 \tag{S7}$$

The decorrelation curve of MMF speckle on strain is verified through experiments and simulations in Fig. S4b.

Our experiments demonstrate successful strain sensing using all-optical computational demodulation, as evidenced in Fig. 2e–f (FBGs) and Fig. 5c (MMFs). Even for high-frequency vibration signals, we have accomplished optical computational demodulation by the fast response of the MMF (Fig. 5d).

Radial deformation response. Similarly, radial deformation introduces external perturbations by increasing the optical path length and locally modifying the refractive index through a mechanism distinct from that of axial strain. The speckle decorrelation curve of MMFs under radial deformation is provided in Fig. S4c.

Torsion response. When an optical fiber undergoes torsion, its propagation characteristics change. However, the basic response mechanisms differ markedly between SMFs and MMFs. Since SMFs support only a single spatial mode, torsion primarily affects the polarization state, which can be modeled using a Jones rotation matrix. Given an input polarization state $\boldsymbol{E}_{\text{in}} = [E_x,\ E_y]^{\text{T}}$, the output after rotation by an angle θ is expressed as:

$$\boldsymbol{E}_{\text{out}} = \begin{bmatrix} \cos\theta & -\sin\theta \\ \sin\theta & \cos\theta \end{bmatrix} \boldsymbol{E}_{\text{in}} \tag{S8}$$

As illustrated in Fig. S4d, when the torsion angle reaches 180°, the polarization state returns to its initial orientation, resulting in the MMF speckle correlation coefficient approaching 1. Deviations from perfect correlation mainly stem from torsion-induced strain variations in the fiber core, creating asymmetry between the 0°–180° and 180°–360° intervals. Moreover, the cladding and coating mitigate the applied torsion, reducing the effective torsion experienced by the optical field and resulting in a correlation period slightly less than the theoretical 180°. Consequently, in our framework, torsion sensing using SMFs is generally limited to an effective range of 0°–90°.

In contrast, MMFs support multiple spatial modes, thus torsion induces more complex effects. Besides rotating the polarization state, torsion introduces non-axisymmetric stress within the larger core, modifying the refractive index distribution and causing intermodal coupling. The propagation constants and phases of individual modes may vary with torsion, leading to substantial changes in the interference pattern. Therefore, the output speckle from MMFs is highly sensitive to torsion, making them promising candidates for high-sensitivity torsion sensing. Furthermore, due to rapid speckle decorrelation with increasing torsion (Fig. S4e), MMF-based torsion sensors can achieve continuous angular coverage over the full 360° range.

Bending response. For an MMF bent with curvature $\kappa = \frac{1}{\rho}$, the modified propagation constants β' are derived from:

$$\beta' \approx \beta - \frac{n_{\text{core}} k_0}{\rho} \langle \psi_i | x | \psi_j \rangle \tag{S9}$$

where $\langle \psi_i | x | \psi_j \rangle$ is overlap integral of the mode field.[3] This confirms that bending preserves the modal structure while introducing phase distortions. Bending also induces transverse compression or tension due to the Poisson effect, characterized by ratio σ, which further modifies the refractive index distribution. The actual effect of bending is scaled by a correction factor $u = 1-(1-2\sigma)\frac{n-1}{n}$, where n is the refractive index of the fiber core. In our model, we set $\sigma = 0.17$, which yields a specific numerical correction to the curvature-induced index modulation and ensures more accurate

modeling of mode evolution under bending. With this simulation framework, the correlation curve of MMF speckle with bending angle is shown in Fig. S4f.

Wavelength response. The propagation constant of the optical field, along with fundamental parameters such as the refractive index of the medium, is inherently wavelength-dependent. While these changes are negligible under single-mode conditions, they become critical in MMFs, including both sensing MMFs and those used as scattering media. As a result, the modal structure and intensity distribution of the optical field in MMFs are highly sensitive to wavelength variations. Even small wavelength shifts can significantly alter light propagation within the MMF, leading to rapid decorrelation of the resulting speckle patterns. This decorrelation process is further accelerated in longer MMFs, as confirmed by numerical simulations shown in Fig. S6. Furthermore, we find that the wavelength shift corresponding to a speckle correlation drop to 0.5 is inversely proportional to the MMF length, i.e., $\Delta\lambda_{1/2} \propto L^{-1}$, consistent with the simulation findings reported by Cao et al. in planar waveguides.[4] In addition, a higher mode count further accelerates correlation decay due to the greater diversity of phase accumulation paths, as illustrated in Fig. S7.

Supplementary Note 4. Cross-sensitivity analysis and multi-parameter sensing

Benefiting from the nonlinear encoding introduced by MMF, the speckle patterns are capable of embedding and transmitting rich information. Specifically, the specklegram generated by the MMF can simultaneously encode multiple physical parameters (including diverse measurands and their spatial distributions along the fiber) through distinct perturbation signatures. This characteristic stems from the complex interplay between modal coupling and interference effects within the scattering medium, where each measurand induces specific modifications to the output specklegram.

Importantly, different measurands (e.g., bending, torsion, frequency offset) influence the speckle in fundamentally different ways, resulting in separable fingerprints within a high-dimensional feature space.[5] Thus, the nonlinear projection enabled by the MMF allows distinct perturbations to be resolved via high-dimensional encoding. In some ideal cases, the increased axial strain path length at different positions of the MMF may result in features that cannot be extracted from the speckle output, although the imperfect manufacturing and mechanical bending of the MMF can improve this confusion.

The encoding of high-dimensional speckle patterns preserves different fingerprints for different types of perturbations, while our nonlinear reconstruction algorithm (Supplementary Note 6) can effectively separate the combined effects. Furthermore, the correlation introduced by the memory effect of MMF maintains the measurable continuity of each perturbation state. These characteristics collectively enable simultaneous multi-parameter sensing with minimized crosstalk, as evidenced in our experimental validation (Fig. 4). We also provide a multiple position multiplexing sensing solution through simulation, with up to 10 sensors utilizing spectrum multiplexing (Fig. S19). However, the multiplexing capability of AOFS-IC is not infinite. The ultimate cross sensitivity resolution of the sensing system fundamentally depends on the information capacity of the speckle pattern and the ability of the calculation method to extract orthogonal features from high-dimensional data space.

Supplementary Note 5. Forward propagation model based on optical field diffraction

The light propagation between two planes (U_1 and U_2) can be described by the Rayleigh-Sommerfeld diffraction integral:[6]

$$U_2(x, y) = \frac{z}{j\lambda} \iint U_1(u, v) \frac{\exp(jkr)}{r^2} \, du \, dv \tag{S10}$$

where λ is the optical wavelength, $k = 2\pi/\lambda$ is the wavenumber, z represents the separation distance between the planes, and $r = \sqrt{(x-u)^2 + (y-v)^2 + z^2}$ denotes the Euclidean distance between points (u, v) on U_1 and (x, y) on U_2. Under the scalar diffraction approximation, Eq. (S10) remains valid when $r \gg \lambda$.

The Rayleigh-Sommerfeld impulse response is given by:

$$h(x, y) = \frac{z}{j\lambda} \frac{\exp(jkr)}{r^2} \tag{S11}$$

With this definition, Eq. (S10) can be reformulated as a two-dimensional spatial convolution:

$$U_2(x, y) = \iint U_1(u, v) h(x-u, y-v) \, du \, dv \tag{S12}$$

Applying Fourier transform properties, we convert the convolution into a multiplication in the frequency domain:

$$U_2(x, y) = \mathscr{F}^{-1} \left\{ \mathscr{F}\{U_1(x, y)\} \cdot \mathscr{F}\{h(x, y)\} \right\} \tag{S13}$$

where $\mathscr{F}$ and $\mathscr{F}^{-1}$ denote the two-dimensional Fourier transform and its inverse, respectively. The Fourier transform of the impulse response $\mathscr{F}\{h(x, y)\}$ corresponds to the optical transfer function $H(f_X, f_Y)$, defined as:

$$H(f_X, f_Y) = \exp\left[jkz\sqrt{1 - (\lambda f_X)^2 - (\lambda f_Y)^2} \right] \tag{S14}$$

where $f_x = -\frac{x}{\lambda z}$, $f_y = -\frac{y}{\lambda z}$ are the spatial frequency coordinates in the Fourier domain.

In optical computing module of AOFS-IC, the total propagation model can be expressed as:

$$I_{\text{out}} = \mathscr{F}^{-1} \left\{ \mathscr{F} \left\{ \mathscr{F}^{-1} \left\{ \mathscr{F} \left\{ I_{\text{in}} \right\} \cdot H_1 \right\} \cdot M \right\} H_2 \right\} \tag{S15}$$

where I_{in} and I_{out} denote the input and output light intensity fields, respectively. H_1 and H_2 are the optical transfer functions corresponding to two diffraction layers with propagation distances of 15 cm and 9 cm, respectively. The phase modulation layer introduced in AOFS-IC is represented by $M(x, y) = \exp[jW(x, y)]$, where $W(x, y)$ denotes the adjustable weight. We implement this model for computer simulations of optical wave propagation, though it is not incorporated into the calibration procedures of AOFS-IC.

Supplementary Note 6. Discussion on training methods

The forward propagation model in diffractive optics (Eq. (S10)) can be interpreted as a simple fully connected process, where the trainable parameters are the weights of the modulation layer. Specifically, the model discretizes the light field propagation process, where the complex amplitude at each input position connects to all output positions through diffraction effects, forming a fully-connected complex-valued weight matrix. The fixed diffraction kernel (Eq. (S11)) defines the initial physical constraints of these connections, while the trainable phase modulation layer dynamically adjusts the strength distribution of these connections by optimizing the weights.

The error backpropagation mechanism is realized through automatic differentiation technology coupled with gradient descent optimization methods. However, gradient-based approaches heavily rely on the precision of the diffraction model and demand extremely accurate optical alignment and system calibration. Therefore, we adopt a model-free nonlinear optimization approach, i.e., genetic algorithm[7] (GA) (Supplementary Note 6). As a population-based metaheuristic, the GA efficiently explores the optimal spatial light modulator (SLM) template within a two-dimensional parameter space through biologically inspired operations, including selection, crossover, and mutation. This derivative-free approach enables robust calibration for optical computing-based fiber sensing without relying on an explicit physical model, making it particularly advantageous in scenarios where exact system modeling is intractable. The global search capability and noise resilience of GA are suitable for our proof-of-concept experiments, as demonstrated by its superior convergence behavior compared to gradient-based methods (Fig. S8).

While the GA provides an efficient and flexible solution for single-layer optimization in our current setup, its scalability may be limited in more complex scenarios, such as joint optimization of multiple modulation layers or multiplexed sensing task demodulation, where slow convergence or suboptimal solutions could arise. To support large-scale quasi-distributed sensing, it is feasible to incorporate diffraction-based neural network training frameworks with backpropagation, thereby accelerating convergence and enhancing training scalability.

Supplementary Note 7. Genetic algorithm for AOFS-IC training

To accurately perform all-optical computational fiber sensing, a calibration process is required. We implement in-situ machine learning using the `GeneticAlgorithmTraining` function (Algorithm 1), which initializes a population (i.e., a set of SLM weight maps) and performs genetic training. The training procedure consists primarily of three functions: `SelectPopulation`, `CrossoverPopulation`, and `MutatePopulation`. The `SelectPopulation` function (Algorithm 2) evaluates and ranks individuals by computing their fitness scores through forward propagation and output intensity measurement, then selects the top performers for reproduction. The `CrossoverPopulation` function (Algorithm 3) generates offspring weight maps by random crossover between parental weight maps, while retaining the excellent parental weights. The `MutatePopulation` function (Algorithm 4) stochastically updates weights according to a predefined mutation rate and a retention ratio.

Algorithm 1: GeneticAlgorithmTraining

```
Input: state_dataset
Output: optimized SLM weight
Initialize population ← [ ];
for individual_id = 1 to INDIVIDUAL_NUM do
    population[ end + 1 ] ← random array of shape (SLM_WIDTH, SLM_HEIGHT)
      with values between 0 and 1;
end
for generation_id = 1 to GENERATION_NUM do
    loss ← SelectPopulation(population, state_dataset);
    index ← indices that sort loss in ascending order;
    population ← population[ index ];
    if min(loss) < STOP_LOSS then
        break;
    end
    population ← CrossoverPopulation(population);
    mutate_rate ← RATE_START + RATE_STEP × generation_id;
    population ← MutatePopulation(population, mutate_rate);
end
```

Algorithm 2: SelectPopulation

```
Input: population, state_dataset
Output: loss for each individual
Initialize loss ← [ ];
for individual_id = 1 to INDIVIDUAL_NUM do
    SLM_weight = 2π × population[ individual_id ];
    WeightLoad(SLM_weight);
    Initialize state_loss ← [ ];
    for state ∈ state_dataset do
        MeasurandControl(state); // Control the measurand externally
        OpticalForward(); // Execute in the optical domain
        output_intensity ← DetectIntensity(); // Read by photodetectors
            (PDs) or cameras
        target_intensity ← MappingFunction(state);
        state_loss[ end + 1 ] ← LossFunction(output_intensity, target_intensity);
            // LossFunction is defined in the Methods
    end
    loss[ end + 1 ] ← RMS(state_loss);
end
return loss
```

Algorithm 3: CrossoverPopulation

Input: population
Output: population after crossover

for $child\_id$ = PARENT_NUM **to** INDIVIDUAL_NUM **step** 2 **do**
  $mother\_id \leftarrow$ random index from 1 to PARENT_NUM;
  $father\_id \leftarrow$ random index from 1 to PARENT_NUM;
  $mask \leftarrow$ random binary mask of shape (SLM_WIDTH, SLM_HEIGHT);
  $population[\,child\_id\,] \leftarrow mask \odot population[\,mother\_id\,] + (1 - mask) \odot population[\,father\_id\,]$;
  $population[\,child\_id + 1\,] \leftarrow mask \odot population[\,father\_id\,] + (1 - mask) \odot population[\,mother\_id\,]$;
end
return $population$

Algorithm 4: MutatePopulation

Input: population, mutate_rate
Output: mutated population

for $mutate\_id$ = SAVE_NUM **to** INDIVIDUAL_NUM **do**
  Initialize $mask$ with shape (SLM_WIDTH, SLM_HEIGHT) ;
  foreach *element* $\in mask$ **do**
    Set to 1 with probability $mutation\_rate$;
    Set to 0 with probability $1 - mutation\_rate$;
  end
  $mutate\_array \leftarrow$ random array of shape (SLM_WIDTH, SLM_HEIGHT) with values between 0 and 1;
  $population[mutate\_id] \leftarrow population[mutate\_id] + mask \odot mutate\_array$;
end
return $population$

Supplementary Note 8. Implementation process of AOFS-IC

The implementation of AOFS-IC architecture consists of two independent stages: one-time training/calibration stage and all-optical repetitive inference/sensing stage.

Training/calibration stage (performed once before measurement). The objective of this stage is to determine the optimal configuration of the scattering medium and the optical diffractive network (ODN), i.e., the phase pattern of the SLM, to establish a mapping from the physical measurand to the output optical intensity. During this initial stage, we indeed employ computers and cameras to capture the speckle patterns for the training of ODN by using digital genetic algorithm. This process is not entirely optical at present. Specifically, the forward propagation is accomplished optically, but the backward propagation is achieved electrically. The all-optical training of ODNs remains a challenging problem in the field of optical computing, and using electronic-assisted methods for training optical physical networks is currently a common practice.[23]

Inference/sensing stage (repeatedly used for sensing). Once the system is calibrated and the ODN is set with the optimized static phase pattern, the system enters the inference stage. It is in

this stage that the sensing and demodulation process is fully all-optical and operates at the speed of light, without any digital processing. The specific workflow is as follows:

- The optical field, which has been modulated by the physical perturbation acting on the fiber sensor, enters the scattering medium (e.g. MMF).
- The scattering medium non-linearly encodes the sensing information into a high-dimensional speckle pattern.
- This speckle field propagates through the fixed, pre-trained ODN.
- The ODN performs an all-optical transformation, directly mapping the speckle information to the optical intensity at one or more predefined locations on the output plane.
- A PD or a PD array directly reads this analog intensity value, which itself represents the magnitude of the measured physical quantity (as in the scheme shown in Fig. 2) or the probability that the sensing quantity falls into a pre-defined category (as in the scheme shown in Fig. 3).

During this inference stage, no speckle imaging, correlation analysis, or any digital computation is required. The tasks of feature extraction and regression/classification are physically accomplished by the propagation of light through the scattering medium and the optimized ODN. The final digitization by the photodetector is an essential interface for any practical sensor system that provides an electrical readout and does not detract from the all-optical nature of the core signal demodulation and computational process.

Supplementary Note 9. Computational paradigm of AOFS-IC

The AOFS-IC architecture embodies a form of optical analog computation that transcends simple transduction. We formally express its operation, which is analogous to a neural network, as follows:

- **Input:** A complex optical field, E_{in}, carrying information encoded by the physical measurand.
- **Hidden Layer (Nonlinear Transformation):** The scattering medium applies a complex, fixed transformation, H_{scatter}, which nonlinearly projects E_{in} into a high-dimensional speckle pattern. This operation, $H_{\text{scatter}}(E_{\text{in}})$, serves as a fixed feature extractor.
- **Output Layer (Linear Transformation):** The diffractive network applies a trainable linear matrix calculation, W_{ODN} (via phase modulation), to the speckle field.
- **Output:** The intensity at a specific spatial location, $I_{\text{out}} = |W_{\text{ODN}} \cdot H_{\text{scatter}}(E_{\text{in}})|^2$, is designed to be proportional to the measurand.

The system is programmed by training W_{ODN} to execute specific sensing tasks such as regression (Fig. 2) and classification (Fig. 3). This paradigm is consistent with implementations of physical neural networks[24] and neuromorphic computing.

The computational essence of AOFS-IC stems from its programmable and universal functionality. The hardware platform (scattering medium and programmable diffractive network) acts as a universal function approximator. Through training and loading different phase patterns, the same physical system can be reconfigured for disparate sensing tasks without hardware changes: it can be configured as a virtual wavelength filter for FBG strain demodulation, instantly reprogrammed

as a virtual polarization analyzer for SMF torsion sensing, or even trained to simultaneously demodulate multiple sensors and physical quantities (Fig. 4). This reconfigurability fundamentally distinguishes it from a fixed transducer.

Furthermore, the system implements complex, nonlinear mappings. The scattering medium nonlinearly encodes subtle input perturbations into a high-dimensional speckle feature space. The ODN then learns and implements an optimal mapping function from these features to the output intensity. This capability allows the scheme to circumvent fundamental sensing range limitations inherent to many simple transducers, as the network can be trained to convert a wide range of speckle changes into predetermined light intensities, with the primary trade-off being between sensing range and accuracy (Fig. 2d).

All-optical computing accurately encapsulates the fundamental reasons for achieving nanosecond-level latency and extremely low power consumption by eliminating photoelectric conversion and electronic processing. In-sensor computing aptly describes the process of offloading and distributing computational tasks from the central processor to the sensor terminal, which significantly alleviates the resource pressure on central nodes. Together, these concepts define sensing information processing paradigm demonstrated by AOFS-IC.

Supplementary Note 10. Computational performance of AOFS-IC

Computation speed. The computational workload in the AOFS-IC system is entirely dominated by optical processing, as no electronic computation is involved during the inference stage. The system exploits the high parallelism inherent in diffractive neural networks, implemented using an SLM with 1920×1080 neurons and a charge-coupled device (CCD) detector or a single PD with 320×256 pixels. In addition, the use of MMFs introduces high-dimensional transformations, effectively performing a vast number of nonlinear encodings. However, the computational capacity of MMFs cannot be directly quantified.

The total optical computation can be decomposed into three main stages. In the modulation layer, each of the $K = 1920 \times 1080$ neurons applies a complex-valued modulation, requiring approximately $6K$ real operations per input (assuming each complex multiplication entails 4 real multiplications and 2 real additions). The diffractive connections to the $M = 320 \times 256$ CCD pixels involve KM complex multiplications and $(K-1)M$ complex additions, corresponding to $4KM$ real multiplications and $2(2K-1)M$ real additions. At the final stage, the detector performs square-law intensity detection, adding an extra $6M$ real operations. Thus, the total number of real optical operations per layer is given by:

$$R = 6K + 4KM + 2(2K-1)M + 6M = 8KM + 4M + 6K \tag{S16}$$

For used hardware configuration ($K \approx 2.07 \times 10^6$, $M \approx 8.2 \times 10^4$), our system reaches 1.36×10^{12} operations for single camera output, demonstrating the massive parallelism of optical computing.

In AOFS-IC, the overall throughput is constrained only by the output frame rate. For the camera-based configuration, the frame rate is 39 fps, corresponding to a computational speed of approximately 53 tera operations per second (TOPs/s). In the single-PD configuration ($M = 1$), while the photodetector operates at a 20 GHz readout rate, its effective bandwidth is limited to 90 kHz, resulting in a practical computational speed of about 2.61 peta operations per second (POPs/s). Enhancing the PD bandwidth could further boost computational ability.

Computation latency. As shown in the main text, the computational latency in the AOFS-IC system primarily arises from the use of scattering media. Although MMFs offer significant advantages over other scattering media (ground glass or integrating spheres), they inherently extend the optical path length, introducing time delays. However, excessively long MMFs provide diminishing returns in terms of high-dimensional mapping capability. This is because, neglecting imperfections such as manufacturing defects, the MMF behaves as a circularly symmetric waveguide. Hence, extending its length does not significantly enhance the ability of modal or polarization evolution, whereas an appropriately chosen MMF length can still offer effective nonlinear mapping capabilities.

For general-purpose AOFS-IC applications, shorter MMFs are typically sufficient. In specific cases such as FBG sensing, the frequency discrimination capability of MMFs—which is proportional to fiber length (Fig. S6)—necessitates the use of longer MMFs to enhance system performance. The experimental setup of all-optical sensing based on FBG uses a 200m MMF, which brings 974 ns delay to AOFS-IC system. Despite this, AOFS-IC maintains an exceptionally low computation latency for all-optical demodulation, remaining below 3 nanoseconds in other configurations.

Energy efficiency. In AOFS-IC, the input optical power is used not only to interrogate the measurand but also to directly drive the optical computing-based demodulation process. The energy efficiency of different sensing schemes depends on the specific system architecture and the employed all-optical demodulation method. FBG-based sensing typically requires a relatively high-power broadband source ($\sim$1 W, $\sim$30 nm), while the reflected power used for demodulation is usually less than 1 mW. Owing to the wide spectral bandwidth of the source, multiple FBGs can be frequency-division multiplexed, thereby improving system-wide energy efficiency. In contrast, sensing units based on MMFs or SMFs require much lower power when using single-frequency sources. In our experimental system, a single-frequency laser input with a power of 0.3 mW is used. We further demonstrate that the classification-based optical computing approach maintains reliable operation even under lower input power condition (40 μW), highlighting its superior power efficiency.

The AOFS-IC system exhibits exceptional energy efficiency. Under ultralow optical power operation (40 μW input power, Fig. S18), the direct energy efficiency of the camera-based scheme (directly used for the optical computational operations) reaches 1.33×10^{18} OPs/J. For the demodulation scheme of a single PD, taking into account energy consumption and signal-to-noise ratio, the direct energy efficiency is calculated as 8.7×10^{18} OPs/J. At the system level, energy efficiency is currently constrained by peripheral device power consumption: SLM (12.5 W), CCD (3.6 W), and PD (<5 W), resulting in a total system power consumption of approximately 21.1 W. Consequently, the resulting system energy efficiency is 3.29 TOPs/J for the CCD-based scheme and 0.15 POPs/J for the PD-based scheme.

While programmable SLM provides flexibility in our demonstrations, practical implementations can employ passive phase plates for enhanced energy efficiency. These plates implement the optimized phase profile through microfabricated surface relief structures, with phase modulation $\Delta\phi(x, y)$ converted to physical height variations $h(x, y) = \lambda\Delta\phi(x, y)/[2\pi\Delta n]$, where λ is the operating wavelength and Δn is the material refractive index difference. These plates can be manufactured through photolithography patterning of silica substrates, typically requiring subwavelength precision phase control. Compared to SLMs, passive plates further eliminate power

consumption and electronic control while maintaining optical performance, though requiring precise initial alignment.

Supplementary Note 11. Sensing performance of AOFS-IC for various measurands

AOFS-IC exhibits highly versatility and can be extended to a wide range of conventional fiber-optic sensing modalities, enabling real-time measurements of various physical measurands. In addition to the FBG-based strain sensing and the high-sensitivity MMF-based strain detection demonstrated in the main text, we further evaluate the generalization capability of AOFS-IC by experimentally assessing several other representative measurands that are convenient to implement in a laboratory setting. We continue to adopt the root mean square error (RMSE) between estimated values and ground truth (GT) as an metric for quantifying sensing resolution.

In Fig. 5 of the main text, AOFS-IC utilize a 5-meter MMF for high-sensitivity strain detection. Building upon this, we employ a 1-meter MMF configuration here to perform strain sensing over a wider dynamic range using a programmable translation stage, as shown in Fig. 9. Axial strain is applied along the fiber axis via the translation stage. The used translation stage has a displacement precision of 2 μm, corresponding to a strain step of 2 με. Our results show a sensing resolution of 0.8825 με within a 0–50 με range (1.77% of the strain range, Fig. S10a), and 5.3616 με within a 0–500 με range (1.07% of the strain range, Fig. S10b). Although MMFs calibrate using piezoelectric transducers can achieve nanostrain-level resolution, this supplementary result highlights that the sensing resolution of AOFS-IC may vary under different calibration conditions.

The linear translation stage is placed horizontally below a section of MMF, providing micrometer level displacement to apply radial deformation. The entire section of MMF is 18 cm. Fig. S11 presents the statistical analysis of radial deformation measurements, comparing the estimated values with GT across two different displacement ranges. AOFS-IC achieves reliable micron-level resolution, with estimation errors of 2.2564 μm over a 100 μm range (2.26% relative error, Fig. S11a) and 4.1420 μm over a 200 μm range (2.07% relative error, Fig. S11b), demonstrating its robustness in detecting subtle physical deformations. Furthermore, we further conduct comparative sensing experiments on deformation displacement to benchmark the demodulation accuracy of AOFS-IC against that of electronic neural networks. As shown in Fig. S16, AOFS-IC achieves an RMSE of 2.2564 μm, which is comparable to that of a diffractive neural network[8] (D2NN) with the same architecture (RMSE is 2.2025 μm), and significantly outperforms a conventional image-based neural network, UNet[9] (RMSE is 5.3579 μm). These results underscore the effectiveness of AOFS-IC in high-precision physical parameter decoding tasks and demonstrate its strong competitiveness with state-of-the-art electronic neural networks.

In addition to the classification-based torsion recognition (as shown in Fig. 2 of the main text), we also implement a regression-based approach that linearly maps optical intensity to torsion angle, enabling continuous and quantitative angle estimation. A fiber segment (MMF or SMF) with a length of approximately 5 cm is used to apply torsion. Utilizing the high sensitivity of MMF, AOFS-IC achieves a high angular resolution of 0.0580° within a narrow torsion range of 0-5° (Fig. S13), corresponding to a relative error of 1.16%. For standard SMF, AOFS-IC yields estimation errors of 0.2186° over a 0–10° range (2.19% relative error) and 1.7862° over a 0–90° range (1.98% relative error), as shown in Fig. S14. Furthermore, we investigate the impact of the

sensing MMF length on performance (Fig. S15). The results reveal that a longer MMF enhances sensitivity, thereby facilitating the ODN detect of minute physical changes.

To further validate the broad applicability of AOFS-IC in bending-based sensing scenarios, beyond the 90° bending measurement demonstrated in the main text, we also evaluate its performance in two additional representative configurations (10° and 180° bending angle estimation) using MMF on the robotic arm. As shown in Fig. S12a, the AOFS-IC system accurately captures bending angles from 0° to 10°, achieving an RMSE of 0.1937° (1.94% of angular range). To assess scalability across a broader angular span, we conduct a wide-range bending experiment covering 0° to 180°, as shown in Fig. S12b. Despite the increased mode coupling and greater dynamic range within the fiber, AOFS-IC maintains strong estimation performance with an RMSE of 5.9074° (3.28% of angular range). The prediction errors remain centered and well-distributed, confirming the robustness of AOFS-IC.

We explore performance of AOFS-IC in decoding spectral shifts caused by strain in frequency-division multiplexed FBG arrays. In simulation, 10 FBGs are placed at distinct wavelengths spanning 10 nm, with each experiencing different applied strain values (Fig. S19a). The corresponding diffractive optical output field is shown in Fig. S19b, while the predicted versus true strain values for each channel are summarized in Fig. S19c. AOFS-IC successfully recovers strain information for all channels with high fidelity. Examples of other combinations are reflected in Fig. S19d–i. The resulting predictions maintain low deviation from GT, highlighting the capacity of AOFS-IC to generalize across complex and independent spectral shift patterns. The overall error distribution, shown in Fig. S19j, yields a mean absolute error (MAE) of 0.5857 $\mu\varepsilon$ and an RMSE of 0.7038 $\mu\varepsilon$, corresponding to 1.66% and 2.01% of the strain range, respectively. Furthermore, regression analysis between predicted and true strain values reveals high linear correlation ($R^2 > 0.99$), as illustrated in Fig. S19k. These results demonstrate that AOFS-IC enables parallel, high-resolution decoding of multiple measurands in a multiplexed FBG array without the need for complex spectral demultiplexing or post-processing.

To further investigate the scalability of AOFS-IC for multiplexed sensing, we extend our simulations to FBG arrays comprising 11 to 18 sensing channels under the same experimental setup (5-layer ODN, 0.08M parameters, 1-meter MMF with 105 μm core diameter and FBG center wavelength interval of 1 nm, etc.) (Fig. S20). The system maintains distinct spatial encoding patterns in the output optical field for each wavelength channel, with predicted strain values closely tracking the ground truth across all multiplexing scales. This demonstrates the robustness and scalability of the architecture in multi-channel strain sensing. Quantitative analysis (Fig. S21) reveals that the strain resolution (RMSE and MAE) remains nearly constant as the number of FBG channels increases from 10 to 15, indicating stable performance. Beyond 16 channels, a gradual increase in RMSE and MAE is observed alongside a rapid decline in R^2. If a strain resolution below 1 $\mu\varepsilon$ is considered the benchmark for acceptable performance, the 16-channel configuration remains effective and reliable. The impact of experimental configuration on the sensing performance is summarized in Fig. S22, revealing the mechanisms and potential routes for further performance improvement.

Finally, we validate the capability of AOFS-IC to perform high-fidelity polarization state reconstruction. Using a 5-layer ODN trained on simulated polarization-dependent optical field distributions, AOFS-IC can directly infer the normalized Stokes parameters $(\mathrm{s}_1, \mathrm{s}_2, \mathrm{s}_3)$ from raw optical intensity images, eliminating the need for conventional polarimetric hardware components. Representative examples of predicted Stokes parameters and GT are presented in Fig. S23a–f, where

the output diffractive fields corresponding to different input polarization states (left) are processed to accurately reconstruct the Stokes vectors (right). Fig. S23g overlays the predicted polarization states (red) onto the Poincaré sphere, showing excellent alignment with GT (blue) across linear, elliptical, and circular polarization modes. The error distribution of predicted Stokes parameters, shown in Fig. S23h, yields an MAE of 0.0046 and an RMSE of 0.0057, corresponding to 0.23% and 0.27% relative errors, respectively. Moreover, regression plots in Fig. S23i demonstrate strong linear correlation between predicted and actual Stokes values across the test set, with $\mathrm{R}^2 > 0.999$ for all components. These results highlight the potential of AOFS-IC as a compact all-optical polarimeter.

Supplementary Note 12. Comparison with classical fiber-optic sensing systems

Explanation on comparisons. First and foremost, it is crucial to recognize that AOFS-IC represents a fundamentally new sensing architecture, not merely an optimization of a specific sensing element. Numerous studies in the literature focus on enhancing the performance of individual sensors through complex structural designs (e.g., specially coated FBGs, polished or side-polished fibers) or advanced algorithms. In our approach, the sensing units are standard, unmodified commercial optical fibers (such as conventional FBGs or standard MMFs). The key advantage of the proposed AOFS-IC architecture lies in its use of all-optical computing to fundamentally transform the signal demodulation process in fiber sensing. The performance comparisons demonstrate that, even with basic sensing units, AOFS-IC can achieve highly competitive overall performance, particularly in dimensions where traditional electronic demodulation systems struggle.

Regarding resolution and sensitivity, most cited works report only the resolution of the sensor itself. Our comparisons show that AOFS-IC achieves comparable resolution. In our system, sensitivity is determined by two factors: 1) The physical sensitivity of the sensing unit: For instance, the fundamental strain sensitivity of an FBG remains 1.2 pm/$\mu\varepsilon$, identical to that in traditional systems. 2) The detection sensitivity of the optical computing system: Theoretically, if the scattering medium can encode infinitesimal optical field variations into distinguishable speckle changes, and our optical network can perfectly decode them, then the detection sensitivity of the system could be extremely high. In practice, the ultimate limit is set by the system noise.

Bandwidth can also be considered from two perspectives: the signal bandwidth perceivable by the sensor, and the response bandwidth of the interrogator (inversely related to demodulation latency). To the best of our knowledge, the physical response bandwidth of the sensing units themselves (e.g., the vibration response bandwidth of an FBG is inversely proportional to its length) can be in the MHz or even GHz range. The AOFS-IC architecture, when based on these conventional sensing units, does not inherently limit the detectable bandwidth of the sensors. Furthermore, AOFS-IC does not introduce complex structures or bandwidth-limiting demodulation methods; it merely establishes a direct mapping from the measurand to the optical field and then to the light intensity, operating at the speed of light. We therefore argue that this architecture does not limit the response bandwidth of the demodulator. The final bandwidth bottleneck in our experiments arises solely from the PD used.

FBG-based strain sensors. To evaluate the performance of AOFS-IC, we first compare key metrics from representative FBG demodulation schemes, as summarized in Table S1. The com-

parison includes sensing accuracy, dynamic range, demodulation speed, power consumption, and multiplexing capability, along with the employed demodulation techniques.

In terms of accuracy, due to the bandwidth limitation of ordinary FBGs and the difficulty of further fine calibration of FBGs, we only achieve a strain resolution of $\mu\varepsilon$. Although Liu et al.[10] reported a resolution of 17.6 nε, it was based on slow wavelength scanning and ultra-narrowband FBGs (50 pm), which limit the dynamic range and speed. Kuse et al.[11] utilized dual-comb interferometry and precise frequency calibration to reach similar performance, but the system complexity is high. In contrast, AOFS-IC follows the standard FBG sensing architecture without requiring additional sensing devices. By introducing optical computing techniques only at the demodulation end, it achieves basic strain sensing, which is sufficient for many practical applications. More importantly, since the demodulation mechanism of AOFS-IC is independent of wavelength scanning or peak detection, its strain dynamic range is inherently scalable, limited only by the source bandwidth and tolerable distortion—though this comes at the cost of reduced accuracy.

The key advantage of AOFS-IC lies in its ultrafast demodulation speed and low power consumption. Most traditional systems rely on optical spectrum analysis (OSA) or spectral scanning, which introduces significant hardware delays (typically in the millisecond level). Even approaches involving neural networks[13,14] can improve resolution via data training, but the digital inference process remains fundamentally limited by the latency and power consumption of electronics. AOFS-IC leverages an upgraded computing architecture based on optical processing. As demonstrated in Supplementary Note 10, its demodulation latency is reduced to below 1 μs (which can be flexibly configured by adjusting the length of the scattering medium, although this depends on the resolution), and the entire inference pipeline can be executed with significantly lower power. Furthermore, AOFS-IC naturally supports multiplexing, leveraging the same wavelength-division multiplexing (WDM) principle as conventional FBG systems (Fig. S19).

MMF-based sensors. MMF sensors offer several inherent advantages, including low cost, high sensitivity, and strong potential for multi-parameter detection. However, conventional MMF-based sensing systems often suffer from complex signal processing pipelines and relatively slow demodulation speeds, limiting their practicality in time-critical or resource-constrained applications.

AOFS-IC provides a uniquely balanced and scalable sensing capability across multiple physical quantities, making it particularly well-suited for real-world deployment (Table S2). Specifically, it achieves a strain resolution of 1.62 nε with a noise floor of 69 f$\varepsilon/\sqrt{\mathrm{Hz}}$, and the sensing range can be extended to the mε level. In addition, the system achieves a deformation sensitivity of 2.26 μm / 200 μm, a torsion prediction resolution of 0.07° / 360°, and a bending detection resolution of 2.8° / 90°. Although certain existing methods surpass AOFS-IC in individual metrics, these typically rely on specialized probes or complex structural designs,[15,19,20,22] which constrain their generalizability, scalability, and robustness. In contrast, AOFS-IC is designed for reliable multi-modal sensing in complex and dynamic environments based on standard MMF, without dependence on custom device structures.

In terms of multi-parameter sensing, AOFS-IC supports more diverse and extensible multiplexing compared to other methods.[17,19,22]Specifically, it simultaneously supports the optical-domain decoding of multiple physical quantities, including strain, deformation, torsion, and bending, across multiple sensing points. To our knowledge, AOFS-IC is the only method to perform multi-parameter demodulation entirely in the optical domain, significantly simplifying system architecture and improving real-time responsiveness. Although temperature sensing has not yet been

demonstrated with AOFS-IC, the inherent thermal sensitivity of MMFs makes it evident that temperature detection is feasible in future implementations.

Notably, AOFS-IC achieves an ultrafast demodulation speed of less than 3 ns, which is several orders of magnitude faster than conventional approaches such as that reported in [18] (36 ms). While traditional MMF sensing systems rarely report energy efficiency, it can be inferred from their reliance on CPUs or GPUs that they incur significant power consumption. In contrast, AOFS-IC avoids this issue by leveraging optical computation. In our experiments, the power consumption of the CCD-based and PD-based implementations are 16.1 W and 17.5 W. The modulator can be further optimized using passive phase plates to improve energy efficiency, and the power consumption of the detector is unavoidable, just like other MMF based sensing schemes.

In summary, AOFS-IC offers superior sensing resolution, faster response, and lower power consumption, while maintaining scalability and multi-parameter detection capability. These features make it highly suitable for the development of next-generation high-performance optical fiber sensing networks.

Supplementary Tables

Table S1. Comparison of various FBG strain sensing schemes.

	Accuracy	Range	Demodu. Speed	Demodu. Power	Multiplex Capability	Fiber Structure	Demodu. Methods
[10]	17.6 nε	100 nε	N.A.	N.A.	✓	Dual narrowband FBG (50 pm)	Tunable-laser scanned and cross-correlation
[11]	34 nε	8 nm	12 ms	N.A.	✓	Standard FBG	Dual-comb interference and moving-window Fourier transform
[12]	1 με	1000 με	N.A.	N.A.	✓	RGO-coated eFBG	FBG optical interrogator
[13]	0.47 pm	2 nm	15 ms	N.A.	✓	Standard FBG	OSA and dilated CNN
[14]	2.8 pm	1 nm	N.A.	N.A.	✓	Specific bandwidth FBG	OSA and CNN
**AOFS-IC (This work)**	2.76 με (3.31 pm)	2.5 mε (3 nm), expandable[a]	0.97 μs, adjustable (only FBG-based)[b]	16.1 W (CCD-based), 17.5 W (PD-based)[c]	✓	Standard FBG	All-optical computational demodulation

Note for FBG-based AOFS-IC:

a. The sensing range is fundamentally scalable beyond 3 nm, as ODN can map speckle patterns to preset intensities without being constrained by the linear region of traditional devices.

b. The 200-m MMF, which introduces the primary latency, is specifically employed here for high-resolution wavelength discrimination and can be optimized for speed in other scenarios.

c. The power of photoelectric conversion equipment is inevitable.

Table S2. Comparison of various MMF sensing schemes.

	**Strain**	**Deform.**	**Torsion**	**Bending**	**Demodu. Speed and Power**	**Multi-parameter Sensing Capability**	**Fiber Structure**	**Demodu. Methods**
[15]	15 με / 22.6 mε	–	–	–	N.A.	×	POF	speckle correlation tracking
[16]	2.9 $p\varepsilon/\sqrt{\text{Hz}}$ / 15 nε	–	–	–	N.A.	×	Standard MMF	Off-axis holographic and signal reconstruction
[17]	–	50 μm / 100 μm	–	1.8° / 88.2°	N.A.	✓	Standard MMF	KNN
[18]	–	–	2° / 360°	–	Speed: 36 ms	×	Standard MMF	ResNet
[19]	∼100 με / ∼35 mε	∼30 nm / ∼10 μm	–	–	N.A.	✓	Designed fiber-end probe	Dual-output layer ResNet
[20]	–	∼0.3 mm / ∼5 mm	–	–	N.A.	✓	Polished fiber and side-and-end coupling	CNN
[21]	–	–	–	1.36° / 360°	N.A.	×	Reflective coating	ResNet
[22]	–	–	0.18° / 360°	0.16° / 5.29°	N.A.	✓	MMF and few-mode multi-core fiber	Dual-output layer CNN
**AOFS-IC (This work)**	1.62 nε (69 $f\varepsilon/\sqrt{\text{Hz}}$) / 0.5 mε	2.26 μm / 200 μm	0.06° / 360°	1.56° / 180°	Speed: 3 ns Power: 16.1 W-17.5 W	✓	Standard MMF	All-optical computational demodulation

Supplementary Figures

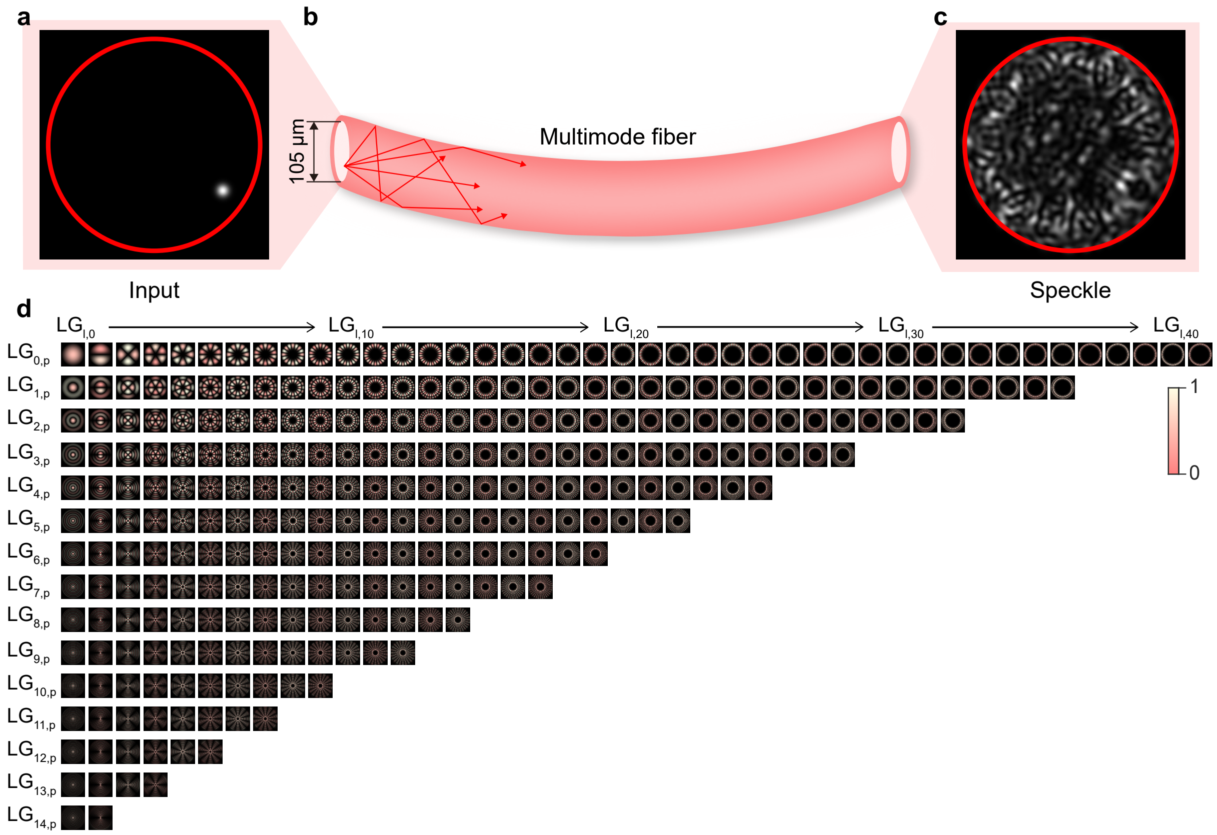

Fig. S1. Numerical modeling and simulation of MMF propagation characteristics. a, Input optical field launched into a MMF. The red circle shows the core of the fiber. **b**, An MMF with 105 µm core. **c**, Speckle patterns of the MMF output field. **d**, Simulation of Laguerre-Gaussian (LG) PIMs in the MMF with a diameter of 105 µm. LG modes are structured light beams carrying orbital angular momentum with helical phase fronts and annular intensity profiles.

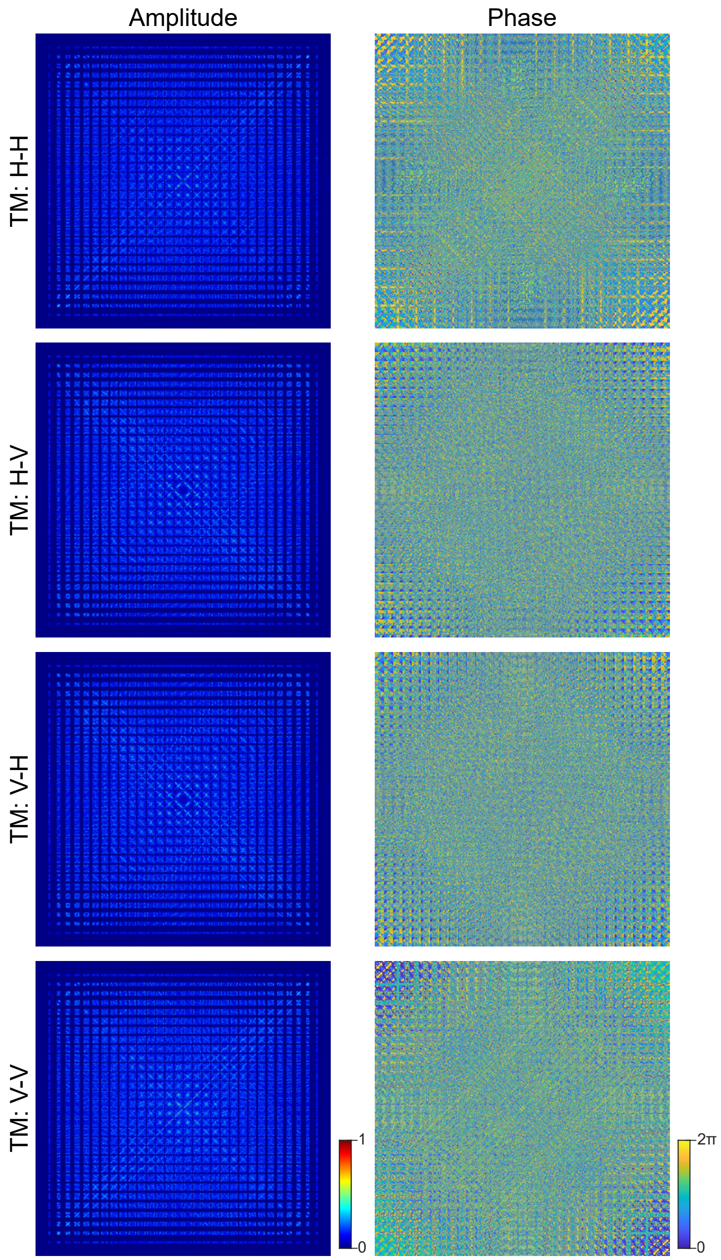

Fig. S2. Amplitude and phase of optical field transmission matrix for a 200 m MMF. First row: horizontal polarization input to horizontal polarization output. Second row: Horizontal polarization input to vertical polarization output. Third row: vertical polarization input to horizontal polarization output. Fourth row: vertical polarization input to vertical polarization output.

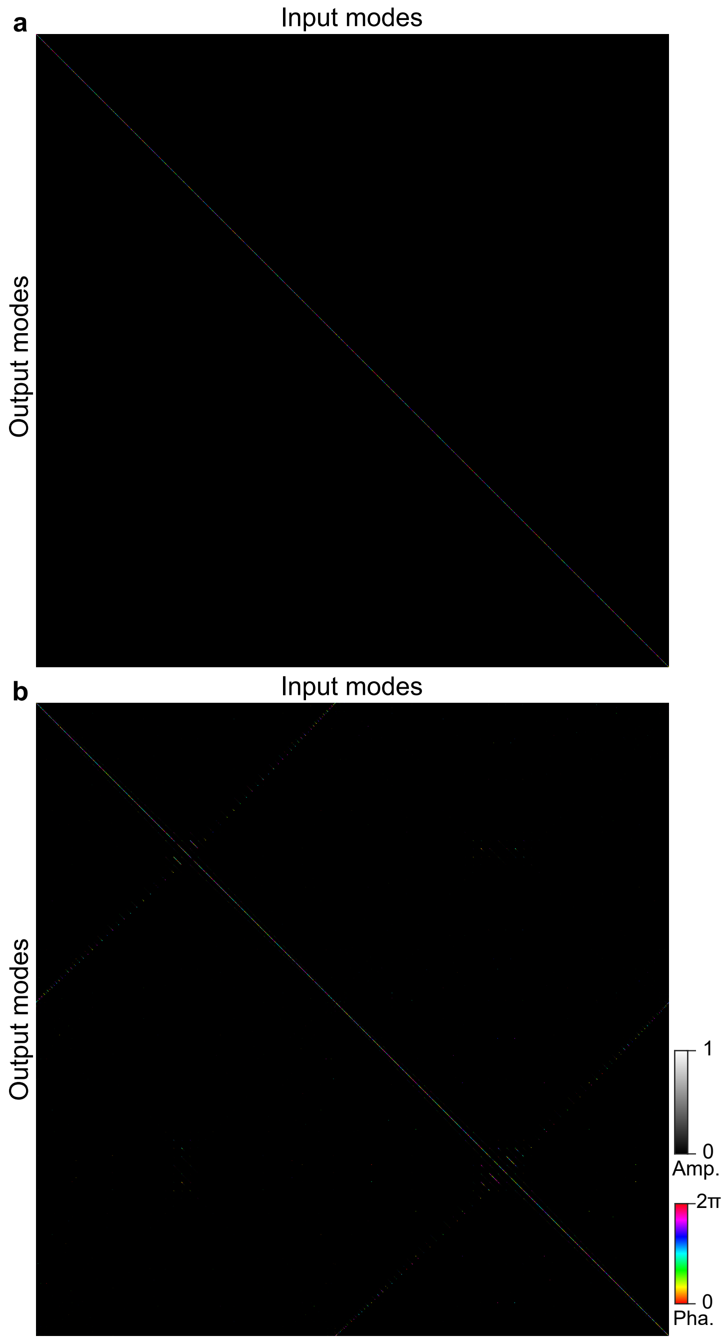

Fig. S3. Modal transmission matrix for straight (a) and bending (b) MMFs. The matrices represent the transmission coefficients from all input modes to all output modes (assuming that all modes are equally excited). In the bending case, mode coupling occurs due to perturbations in the fiber geometry, as described in the theoretical model proposed by Plöschner et al.[3]

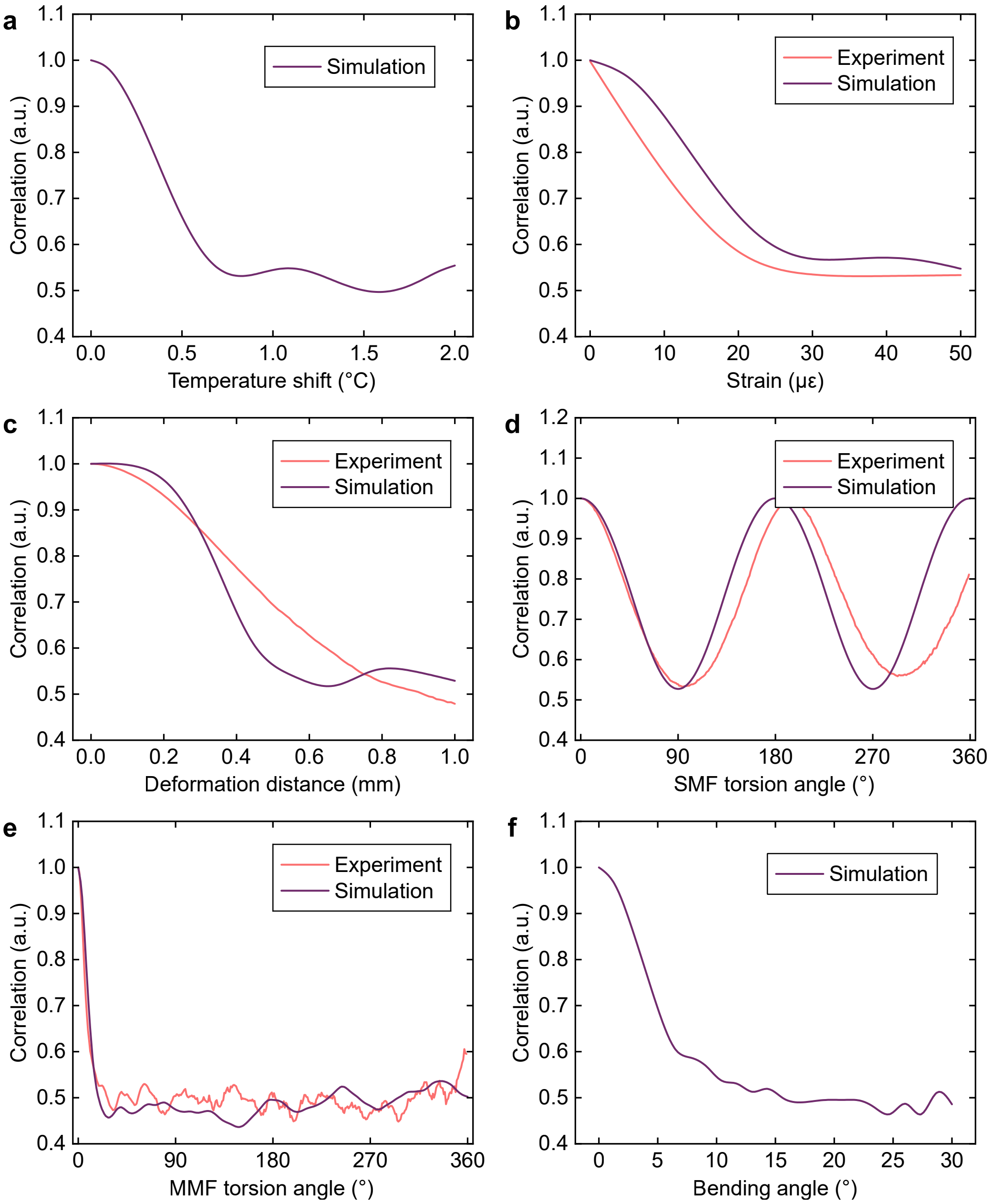

Fig. S4. Simulated and experimental speckle decorrelation under various physical perturbations. NPCC is used to quantify the correlation between speckle patterns. **a**, Simulated decorrelation of MMF speckle patterns due to temperature changes (0–2°C), showing rapid decline in correlation within 2°C. **b**, Experimental and simulated speckle correlation results for axial strain (0–50 με), demonstrating sensitivity to strain-induced changes in optical path length. **c**, Speckle decorrelation under radial deformation (0–1 mm), where external pressure alters the local refractive index and modal distribution. **d**, Speckle correlation under torsion in SMFs (0–360°), exhibiting 180° periodicity due to polarization rotation. Slight asymmetries arise from strain effects and imperfect mechanical coupling. **e**, Experimental and simulated torsion-induced decorrelation in MMFs, showing rapid correlation decay in 30° range without periodicity, owing to intermodal coupling and refractive index modulation. **f**, Simulated bending response of MMF under angular deflection from 0° to 30°, where bending-induced phase perturbations and Poisson effects lead to a gradual reduction in correlation.

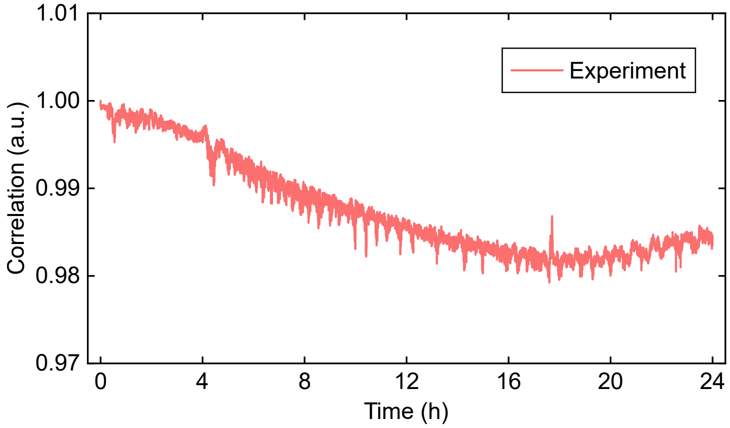

Fig. S5. Long-term stability of the speckle pattern in AOFS-IC. Over a 24-hour measurement period, the speckle pattern exhibits only minor variations caused by environmental factors such as ambient temperature drift (gradual decline and partial rebound) and mechanical perturbations (occasional sharp spikes). Since the stability of the speckle directly affects demodulation accuracy, maintaining a high correlation is essential. The correlation coefficient remains consistently above 0.98, underscoring the robustness and reliability of AOFS-IC for continuous fiber-based sensing.

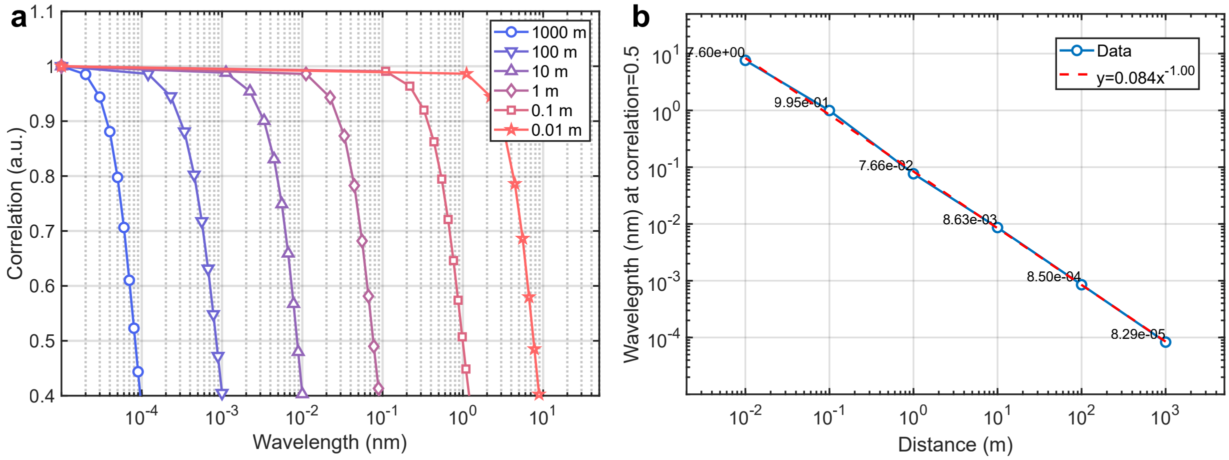

Fig. S6. Wavelength-induced speckle decorrelation in MMFs of varying lengths. a, Simulated speckle pattern correlation as a function of wavelength shift for MMFs of different lengths, ranging from 0.01 m to 1000 m. Longer MMFs exhibit faster decorrelation in response to wavelength variation (i.e., requiring smaller wavelength shifts to reach the same decorrelation level). **b**, Wavelength shift corresponding to a correlation coefficient of 0.5 plotted against MMF length on a log-log scale. The data follows an inverse relationship, with a fitted trend line showing a power law of $y = 0.084x^{-1.00}$, confirming that the sensitivity to wavelength increases proportionally with fiber length.

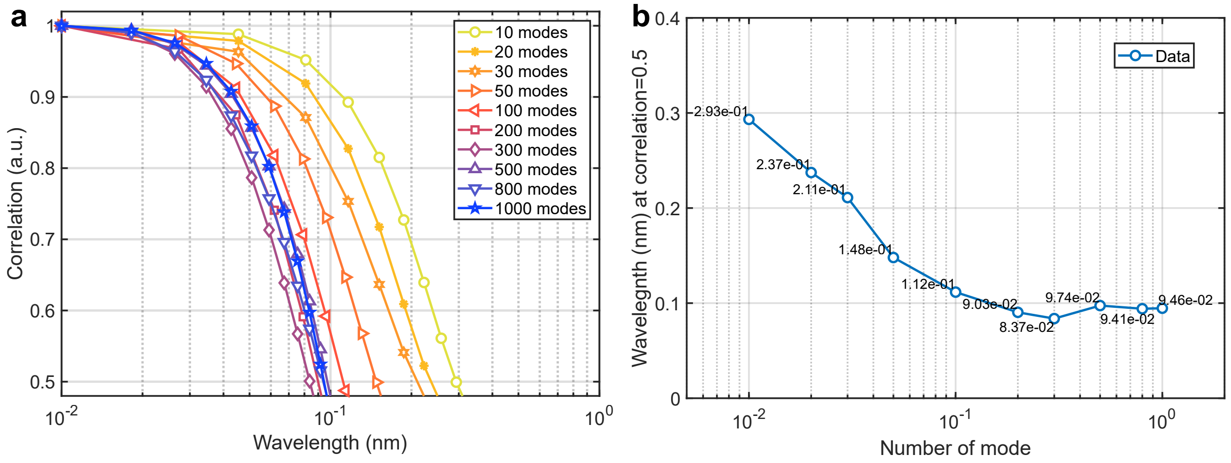

Fig. S7. Wavelength-induced speckle decorrelation in MMF with different numbers of modes. a, Simulated speckle pattern correlation as a function of wavelength shift for different numbers of guided modes in MMFs, ranging from 10 to 1000 modes. Speckle patterns generated by MMFS supporting more modes decorrelate more rapidly with wavelength variation, indicating enhanced spectral sensitivity. **b**, Wavelength shift corresponding to a correlation coefficient of 0.5 plotted against the number of modes. A clear decreasing trend is observed, with spectral sensitivity improving as the number of modes increases.

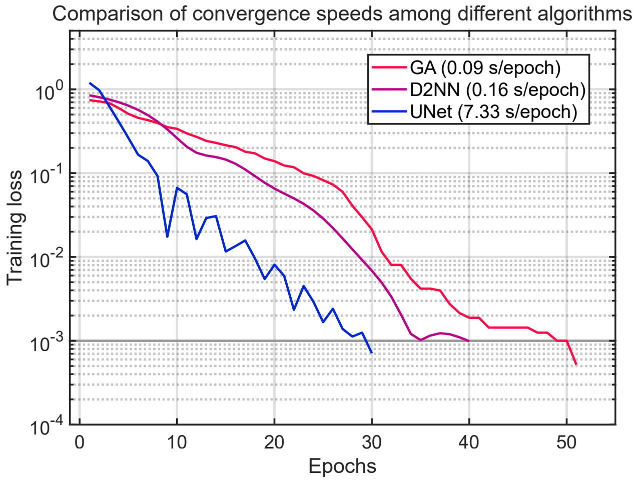

Fig. S8. Training speed comparison of different optimization methods for sensing tasks. We compare the training loss convergence of three optimization methods: our method (GA), diffractive deep neural network (D2NN), and UNet. D2NN represents a numerically simulated counterpart of our physically implemented optical computing system, while UNet serves as a classical deep learning benchmark widely used in image processing. All methods are evaluated using the same loss function and dataset to ensure a fair comparison. D2NN achieves relatively fast convergence, reducing the training loss below 10^{-3} within 40 epochs, with a moderate computational cost of 0.16 s per epoch (total: 6.4 s). GA requires more epochs to converge (51 epochs), but its significantly lower per-epoch time (0.09 s) results in a shorter overall training time of just 4.59 s. In contrast, UNet incurs a substantially higher computational cost (7.33 s per epoch), leading to a total training time of 219.9 s. This comparison demonstrates the superior efficiency and practicality of the GA-based training method for optical computing in sensing applications.

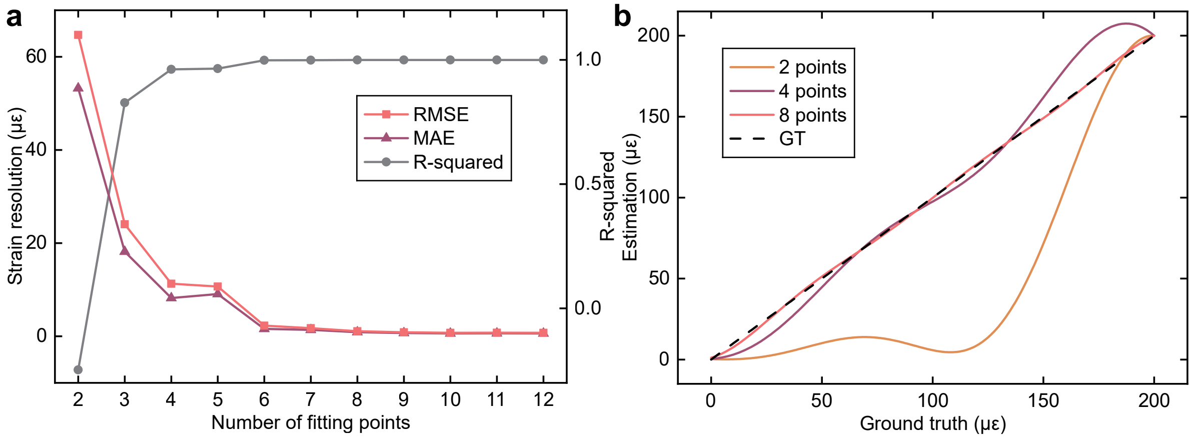

Fig. S9. Impact of the number of fitting points on predicted strain resolution. **a**, The RMSE, MAE and R-squared of the ODN model are plotted as functions of the number of calibration (fitting) points. Both RMSE and MAE decrease rapidly as the number of calibration points increases from 2 to 6, indicating that additional calibration points significantly enhance strain resolution and model generalization. Beyond six calibration points, the errors approach saturation, suggesting that the ODN can achieve stable and high-accuracy strain prediction with only a limited number of calibration points. **b**, Predicted strain versus ground truth for different calibration points. Increasing the number of points enhances linearity and reduces overfitting between calibration regions.

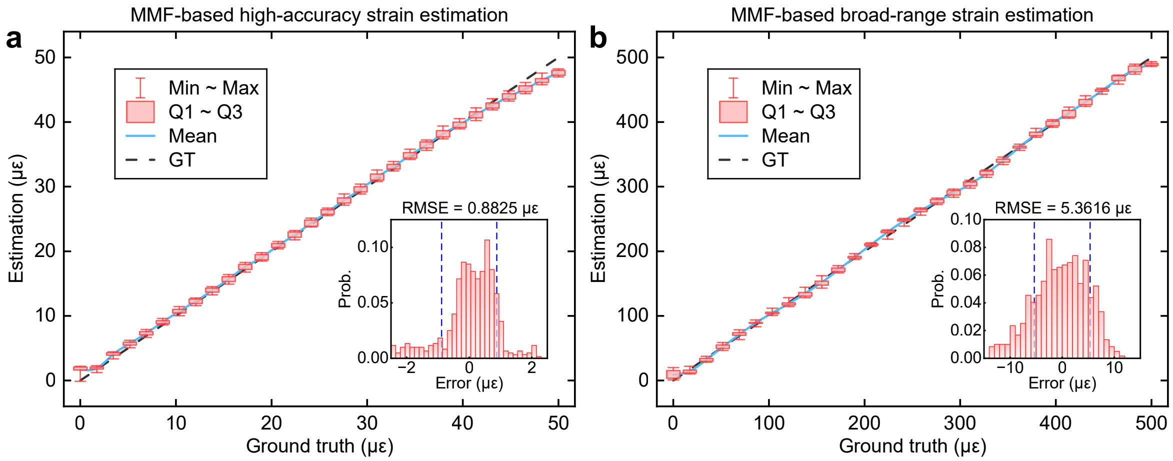

Fig. S10. Strain sensing performance of MMF-based system calibrated using a programmable translation stage under different strain ranges. **a**, Estimation results under a 0–50 με strain range. The predicted mean values closely follow GT (dashed line), with an RMSE of 0.8825 με. The error distribution shown in the inset indicates most estimations fall within ±1 με, constrained by the 2 με calibration resolution of the translation stage. **b**, Estimation results under a broader 0–500 με strain range, with a higher RMSE of 5.3616 με. A comparison with the nε-level resolution demonstrated in Fig. 5 of the main text highlights that the actual sensing performance is highly dependent on the accuracy of the calibration process.

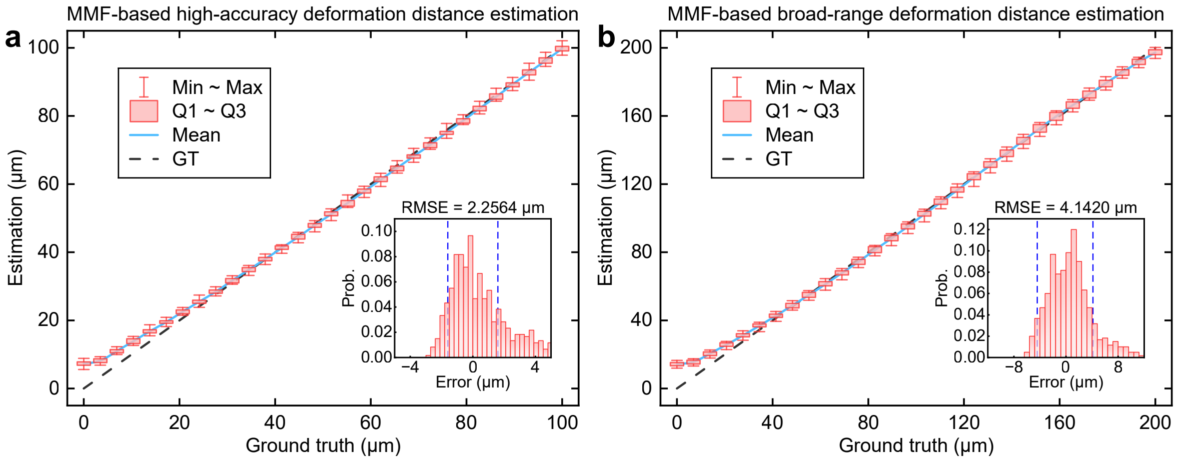

Fig. S11. Deformation sensing performance under different displacement ranges. a, Estimation results over a displacement range of 0–100 μm. The predicted values closely follow GT (dashed line), with an RMSE of 2.2564 μm. The inset shows a histogram of estimation errors, indicating that most predictions fall within ±2 μm. **b**, Estimation results over a larger range of 0–200 μm, yielding a slightly increased RMSE of 4.1420 μm. The broader error distribution shown in the inset reflects the reduced accuracy under larger deformation. The similarity in estimation curves and error patterns suggests that the performance is partially limited by imperfections in the calibration process.

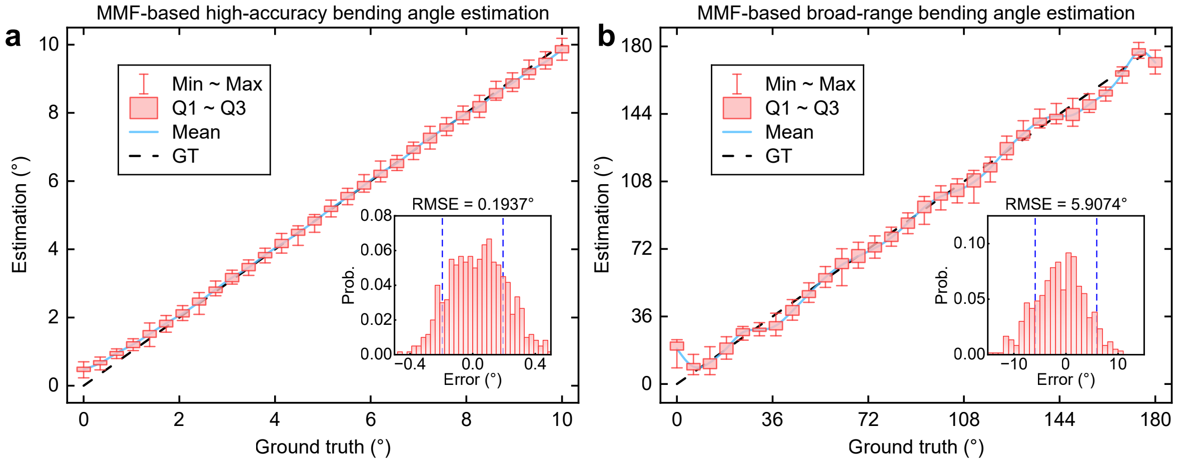

Fig. S12. Bending angle estimation using MMF-based sensing. a, Bending angle estimation over a narrow range of 0–10°. The AOFS-IC shows excellent linearity and resolution, with an RMSE of 0.1937°, highlighting its potential for precision sensing. **b**, Estimation performance over a broad range of 0–180°. Despite the extended range, the system maintains reliable estimation capability, with an RMSE of 5.9074°. The nonlinearity of the estimation results on a large scale primarily arises from the rapid decorrelation of the speckle pattern, which could be mitigated by optimizing MMF routing configurations or increasing the training epochs to improve fitting ability. Insets in both plots show error distributions centered around zero, validating consistent and repeatable decoding across varying angular spans.

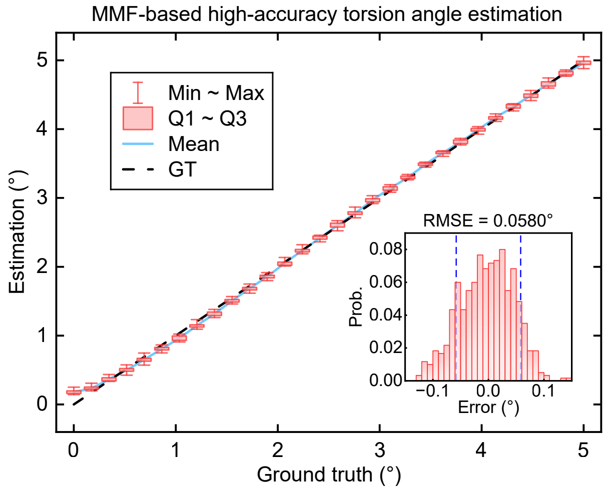

Fig. S13. Continuous torsion angle estimation using MMF-based sensing. While the main text demonstrates a classification-based approach for torsional state recognition in MMFs, here we further present regression-based results for continuous torsion angle estimation. The predicted mean values align well with GT (dashed line), achieving an RMSE of 0.0580°. The error histogram in the inset shows a narrow distribution, with most estimation errors falling within ±0.05°, confirming the high accuracy of the regression-based sensing method.

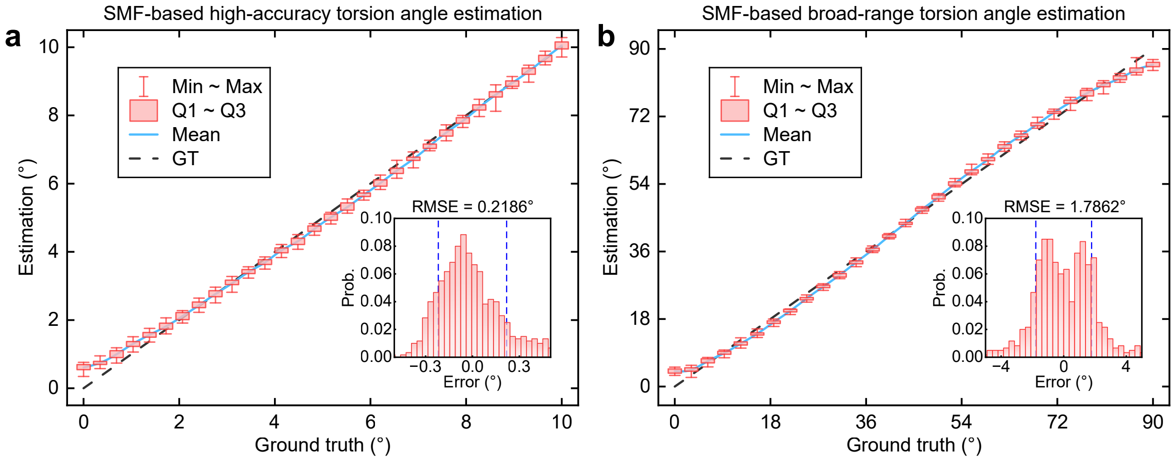

Fig. S14. Continuous torsion angle estimation using SMF-based sensing. **a**, Estimation performance over a small torsion range of 0–10°, achieving am RMSE of 0.2186°. The predicted values closely follow GT, and the inset error histogram shows that most estimations fall within ±0.2°. **b**, Estimation over a broader torsion range of 0–90°, yielding an increased RMSE of 1.7862° due to the expanded dynamic range. The error distribution becomes wider (Most errors are concentrated within ±2°), but the predicted values still generally track GT well. These results demonstrate the feasibility of continuous torsion measurement using standard SMFs across different angular ranges.

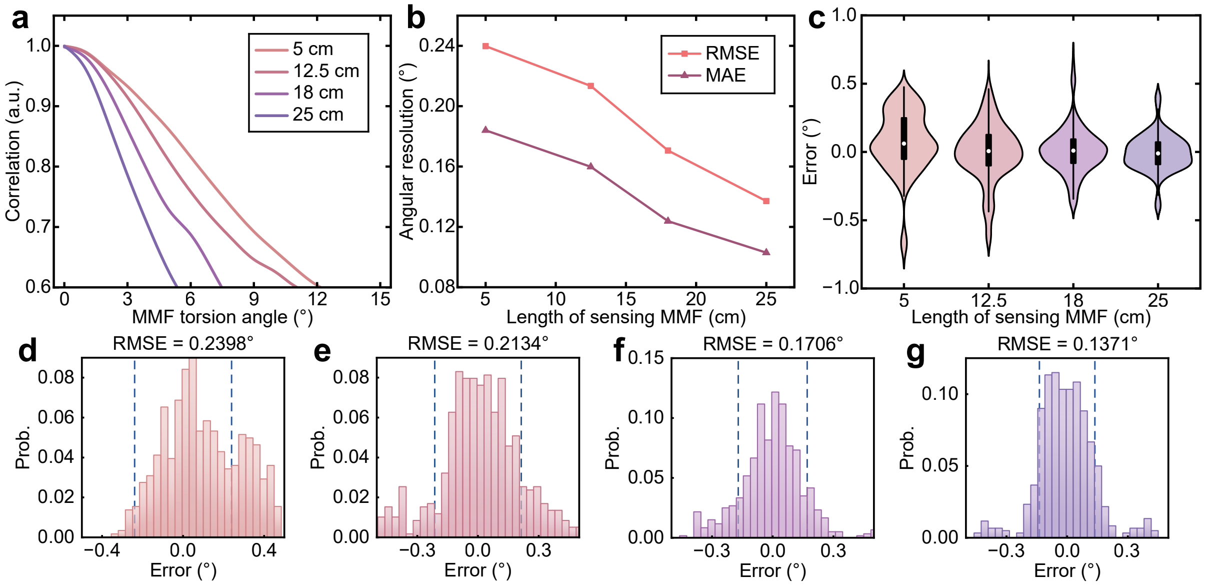

Fig. S15. Impact of sensing MMF length on torsion sensing performance. a, Speckle pattern correlation as a function of MMF torsion angle for different sensing MMF lengths (5 cm, 12.5 cm, 18 cm, and 25 cm). Longer fibers exhibit faster decorrelation with increasing torsion angle. **b**, Angular resolution (RMSE and MAE) plotted against MMF length, showing improvement with increasing fiber length. **c**, Violin plots of angle estimation errors for different MMF lengths, revealing reduced error dispersion with increasing length. **d–g**, Histograms of estimation errors for MMF lengths of 5 cm, 12.5 cm, 18 cm, and 25 cm, respectively, with corresponding RMSE values confirming enhanced accuracy for longer fibers.

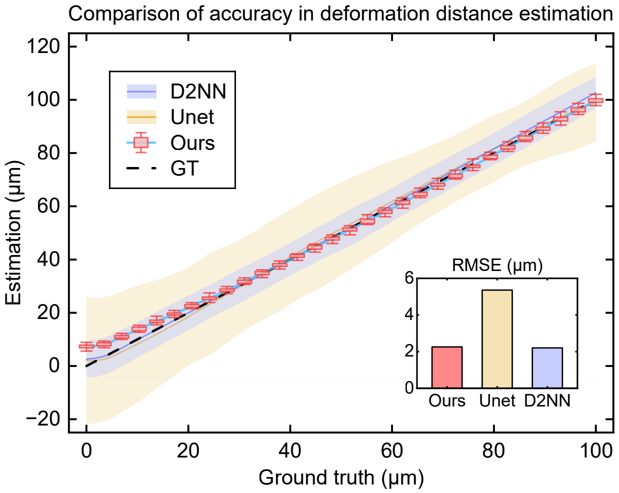

Fig. S16. Comparison of accuracy in deformation distance estimation. Predicted deformation distances are plotted for three different models: the diffractive neural network (D2NN; blue band, RMSE = 2.2025 μm),[8] the UNet-based image network (yellow band, RMSE = 5.3579 μm),[9] and our AOFS-IC approach (red box plots, RMSE = 2.2564 μm). The shaded bands represent the error ranges. Both D2NN and our method show the best agreement with GT (black dashed line) and achieves the lowest estimation error. Inset: Quantitative comparison of RMSE using different methods, indicating that our approach can achieve the lowest RMSE.

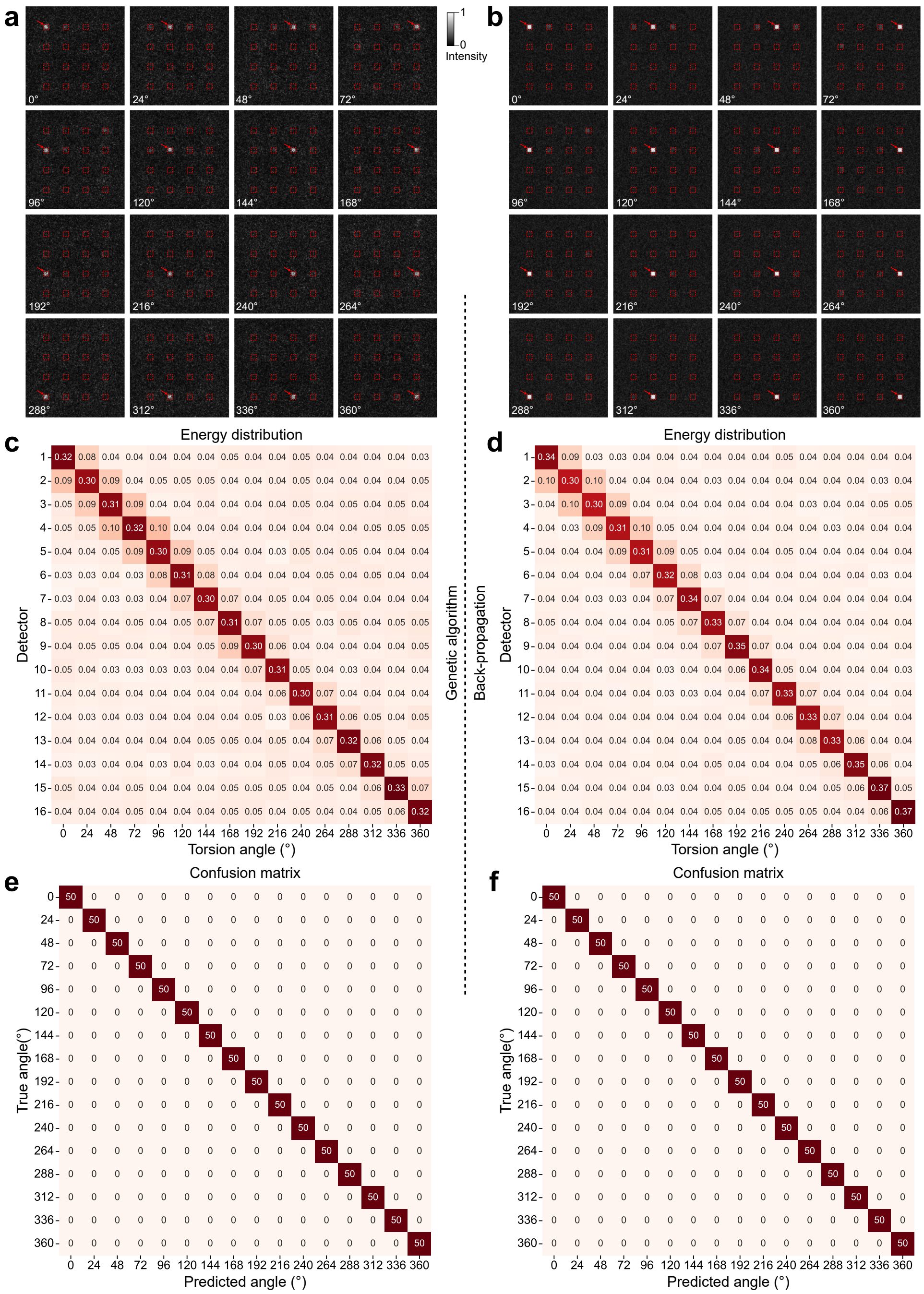

Fig. S17. Torsion angle classification results under two training algorithms. a–b, Output intensity images captured under 16 discrete torsion angles (24° steps from 0° to 360°) using single layer ODN. The red dashed boxes indicate the detectors (within a 4 × 4 array) that register the maximum intensity at each angle. (**a**) shows results obtained using GA (left column), while (**b**) shows results trained via gradient-based back-propagation (right column). **c–d**, Corresponding energy distributions across detectors for each torsion angle. Both training methods achieve comparable signal-to-noise ratios, as reflected by the concentration of energy along the diagonal. **e–f**, Confusion matrices for classification results over 800 testing samples (50 samples per class). Both achieve perfect accuracy across all 16 classes. The experimental RMSE is 6.93° (1.93% of the angular range), limited by quantization noise. This confirms that increasing the number of classification states across the entire angle range can improve accuracy of AOFS-IC.

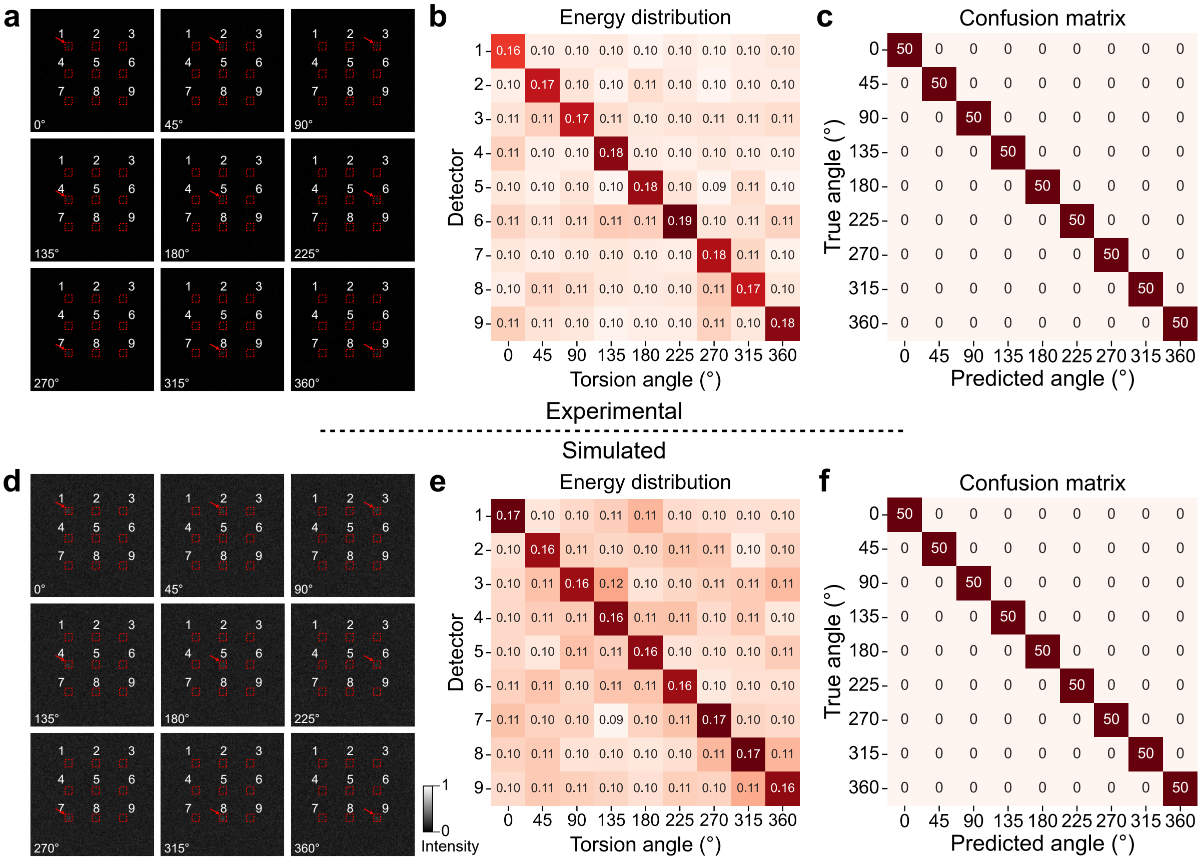

Fig. S18. Robust torsion angle classification under low-light conditions using a single layer diffractive network. a–c, Experimental results with 9 discrete torsion angles (45° steps from 0° to 360°) under a low input optical power of 40 μW. The red dashed boxes in (**a**) mark the detectors in the 3 × 3 region with maximum response at each torsion angle. The corresponding energy distribution across detectors (**b**) and confusion matrix (**c**) confirm robust classification performance with ∼3 dB SNR and 100% classification accuracy. **d–f**, Simulated results under the same configuration, also showing highly concentrated energy distributions (**e**) and perfect classification (**f**), demonstrating strong agreement with experimental results. Both experimental and simulation results highlight the significant energy efficiency and robustness of AOFS-IC under extreme low-light conditions.

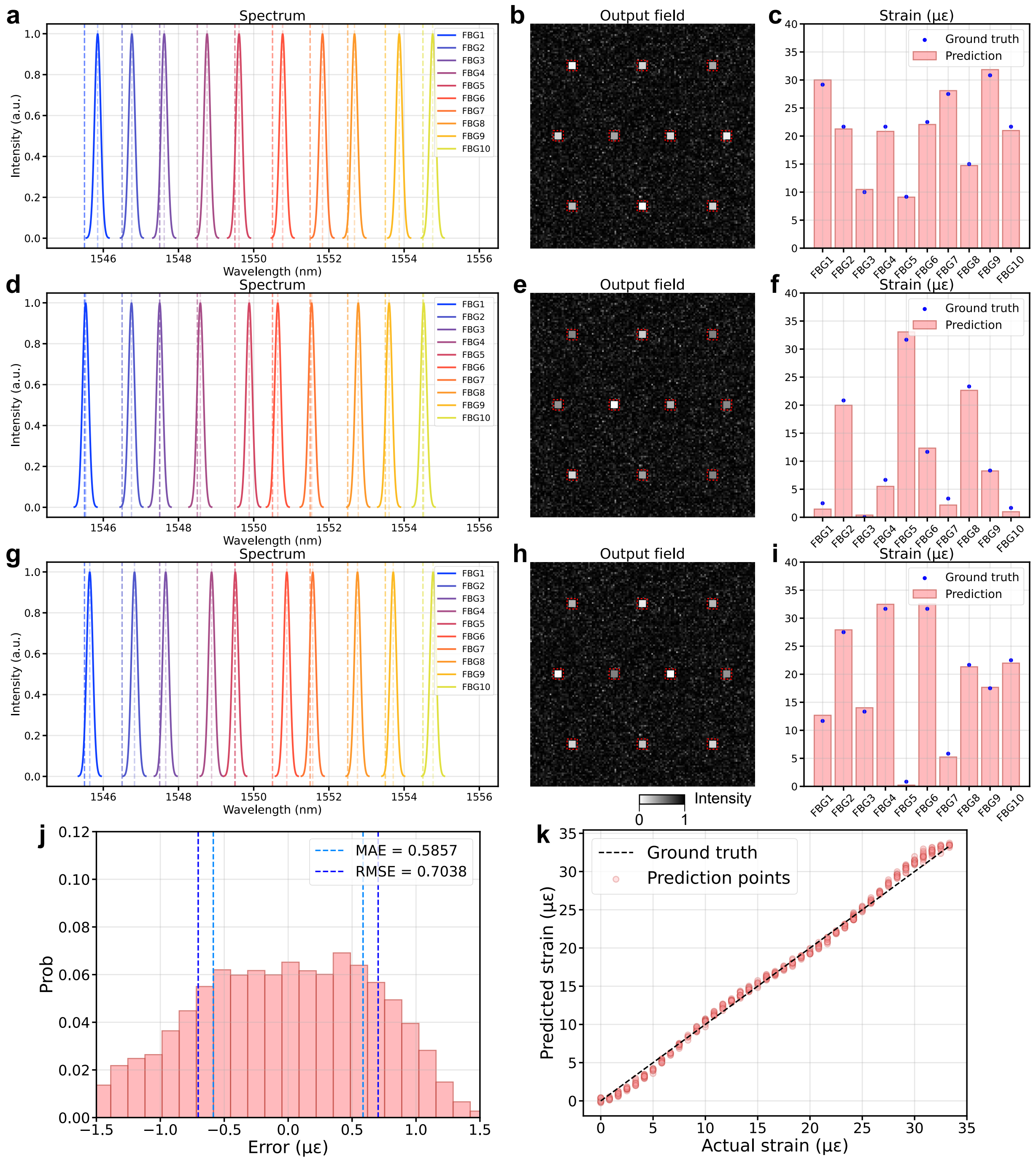

Fig. S19. Multiplexed FBG-based strain sensing using AOFS-IC with a 5-layer diffractive network. a–c, Simulated results showing the reflection spectra of 10 FBGs under different applied strains (**a**), the corresponding diffractive optical output field (**b**), and predicted versus true strain values for each FBG (**c**). **d–i**, Additional simulation results with different strain combinations. **j**, Error distribution of the strain prediction shows an MAE of 0.5857 με (1.67% of strain range) and an RMSE of 0.7038 με (2.01% of strain range). **k**, Across the full measurement range, the predicted strain exhibits a strong linear correlation ($R^2 > 0.99$) with GT. These results indicate that AOFS-IC can effectively learn strain-induced spectral shifts from the multiplexed FBG signals and achieve high-resolution, parallel decoding of all 10 channels. This further implies the potential of AOFS-IC as a compact and low-power platform for high-speed all-optical spectrometers.

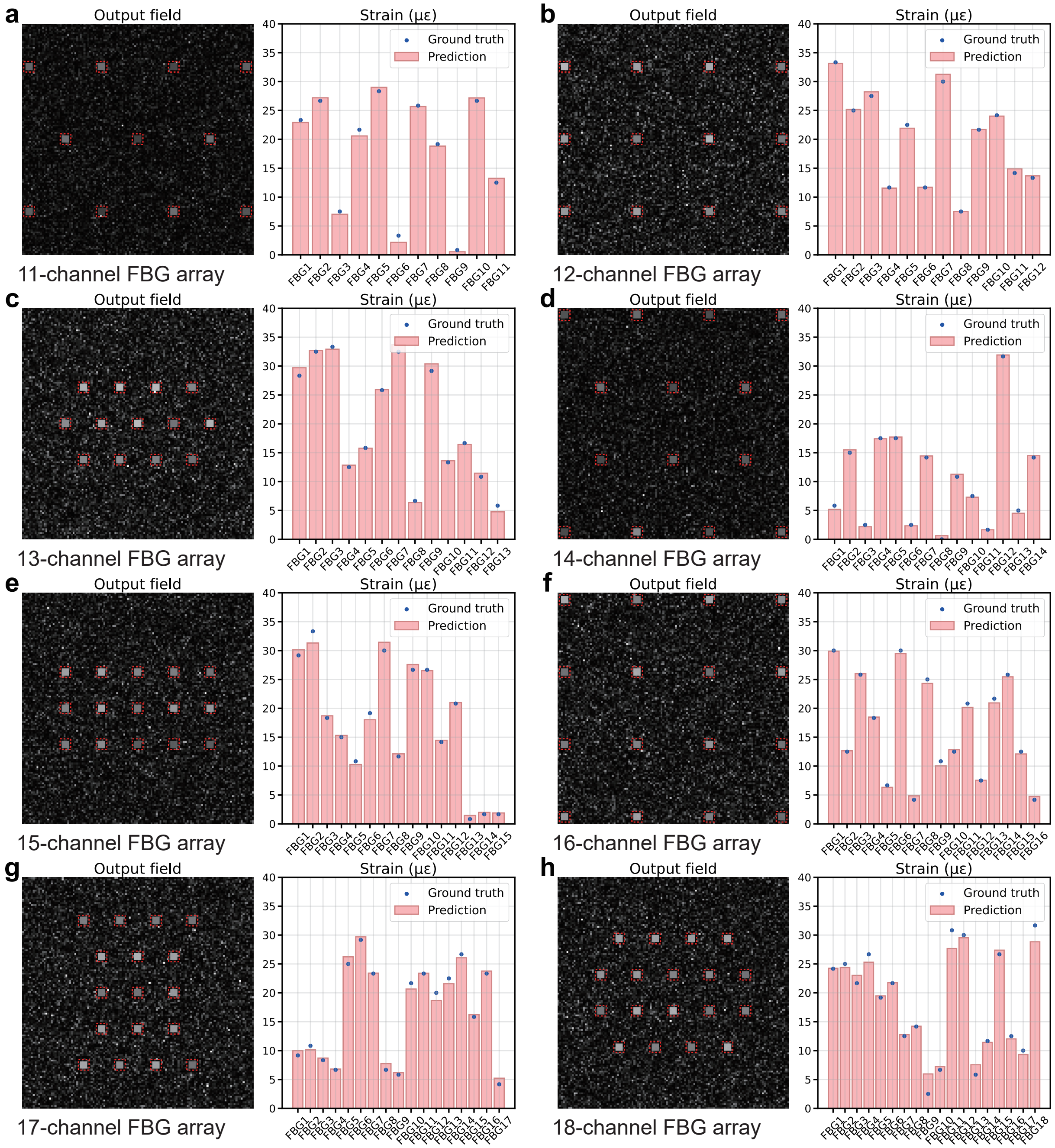

Fig. S20. Multiplexed sensing performance of AOFS-IC with different numbers of FBG channels. a–h, Simulated output optical fields (left) and the corresponding predicted and real strain values (right) for FBG arrays with 11–18 sensing channels. Each output optical field displays distinct spatial encoding patterns, where each receiving region corresponds to a specific FBG wavelength channel. The predicted strain values closely follow the ground truth across all multiplexing scales, demonstrating the scalability and robustness of the AOFS-IC architecture for multi-channel strain sensing. Even as the number of sensing channels increases, the system maintains acceptable reconstruction accuracy and stable optical field responses to a certain extent.

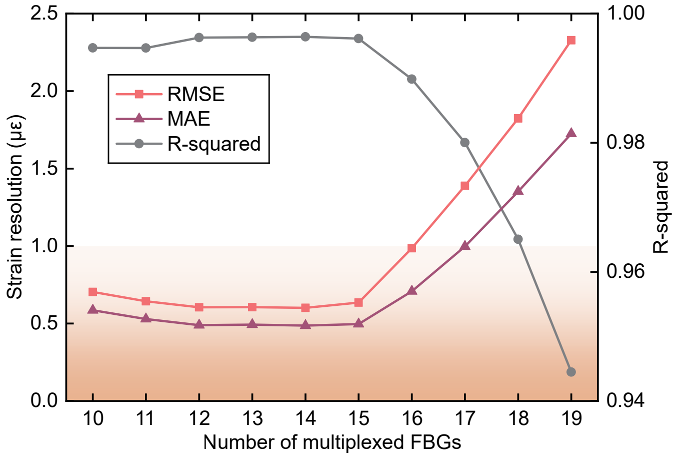

Fig. S21. Quantitative evaluation of multiplexed sensing performance with different numbers of FBG channels under the same ODN configuration. The strain resolution (RMSE and MAE, left axis) and fitting accuracy (R^2, right axis) of the AOFS-IC are plotted as functions of the number of multiplexed FBGs. The strain resolution remains nearly constant as the number of FBG channels increases from 10 to 15, indicating stable sensing performance. Beyond 16 channels, both RMSE and MAE gradually increase while R^2 rapidly decreases, reflecting the performance degradation due to inter-channel crosstalk and reduced speckle contrast. If a strain resolution below 1 με is taken as the criterion for acceptable multiplexed sensing, the 16-channel configuration can still be considered effective and reliable.

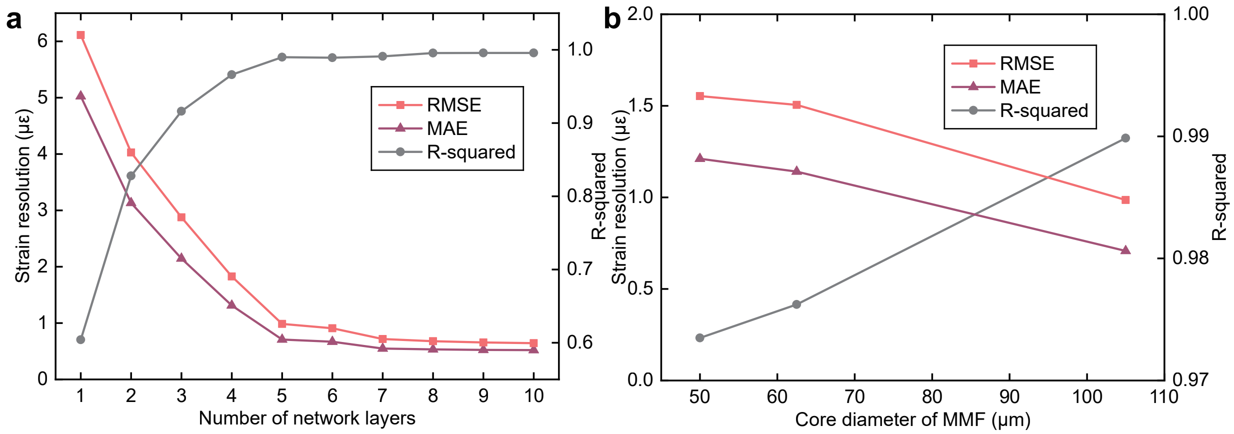

Fig. S22. Quantitative evaluation of the multiplexed sensing performance of AOFS-IC with network layers and MMF core diameter. a, Dependence of strain resolution (RMSE and MAE, left axis) and fitting linearity (R^2, right axis) on the number of diffractive network layers. Increasing the network depth significantly improves strain decoding accuracy and fitting effect. **b**, Dependence of strain resolution and R^2 on the core diameter of MMF. Larger core diameters yield higher R^2 values and lower strain errors, indicating enhanced information capacity of speckle patterns. Therefore, the performance limitations of this multiplexed sensing scheme primarily arise from the information capacity limit of speckle patterns transmitted through MMFs and the constraints of ODN learning ability.

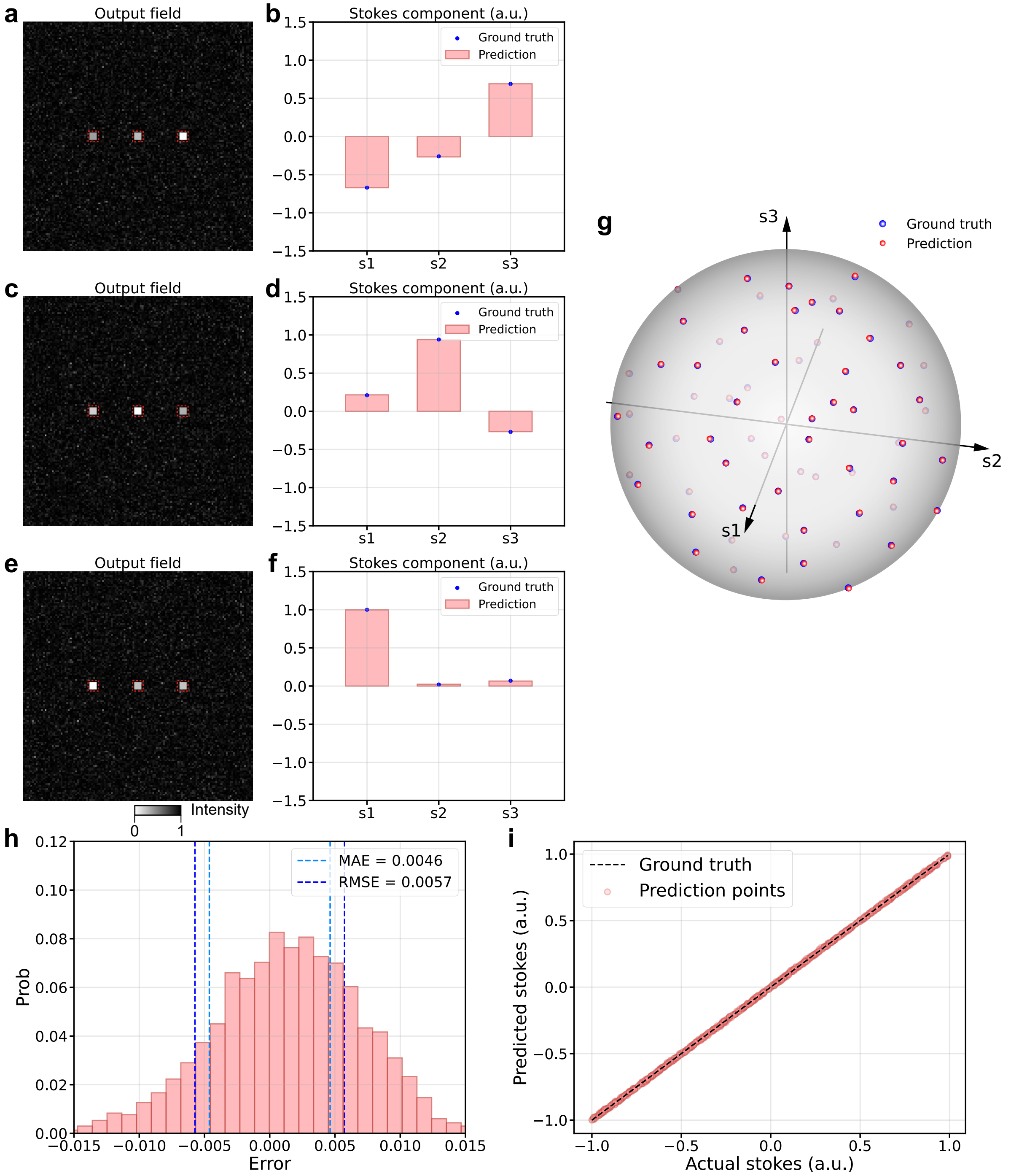

Fig. S23. Polarization state reconstruction using AOFS-IC with a 5-layer diffractive network. **a–f**, Simulated output optical fields (**a**, **c**, **e**) and the corresponding predicted and ground-truth Stokes parameters (**b**, **d**, **f**) for representative polarization states. **g**, Reconstructed polarization states (red) overlaid on the Poincaré sphere, showing excellent agreement with GT (blue) across a wide variety of input polarization conditions. **h**, Error distribution of the predicted Stokes components, with an MAE of 0.0046 (0.2% relative error) and an RMSE of 0.0057 (0.29% relative error). **i**, Predicted versus actual Stokes parameters over the full test range, demonstrating high-fidelity reconstruction with strong linear correlation ($R^2 > 0.999$). These results confirm the capability of AOFS-IC to learn and decode the full polarization state with high accuracy from diffractive optical measurements.

Supplementary Videos

Supplementary Video 1. This video demonstrates the real-time sensing capability of AOFS-IC for monitoring joint angles of a 3-DOF robotic arm using a single MMF. In the demonstration, the robotic arm is sequentially moved to several randomly selected configurations. For each configuration, the pre-trained AOFS-IC system directly analyzes the optical signals transmitted through the MMF and outputs the corresponding joint angle information in real time. As shown in the video, the system exhibits rapid sensing performance, with latency primarily limited by the frame rate of the validation camera. In practical implementations, this bottleneck can be significantly alleviated by employing PD array (with the same number of PDs as DOF of the robotic arm), enabling true high-speed operation. After each movement, the arm is briefly paused to allow the predicted angles to be clearly observed and validated. The observed errors (primarily due to limitations in the external fiber arrangement) are consistent with the error range reported in the main text (RMSE $< 2°$).

Supplementary Video 2. This video demonstrates a proof-of-concept closed-loop control task using AOFS-IC. In this setup, the robotic arm is programmed to perform a simple grasping task. The AOFS-IC system is trained in advance to infer joint angle information from the received optical intensity signals in real time. During execution, the AOFS-IC provides continuous sensing feedback that is used to iteratively control the stepwise motion of the robotic arm. Specifically, based on the decoded joint angles, the system decides whether to issue a step-up or step-down control signal to reach the desired configuration. The robotic arm successfully completes the grasping task under AOFS-IC guidance, validating the feasibility of using optical neural networks for basic closed-loop robotic control.

Supplementary Video 3. This video illustrates the evolution of the strain–intensity mapping when the ODN is trained with varying numbers of calibration points within the 0–200 $\mu\varepsilon$ range. When only two calibration points are used, the model exhibits clear overfitting, with the predicted strain values deviating significantly between the training points. Increasing the number of calibration points to four substantially reduces overfitting, yielding smoother transitions and improved alignment between predicted and true strain. When eight calibration points are employed, the predicted strain demonstrates a nearly linear correspondence with the real strain across the entire range, confirming the strong generalization capability of the ODN. More quantitative results are presented in Fig. S9. The results under different calibration conditions intuitively confirm the fitting and generalization behavior of ODN, which learns smooth continuous mappings constrained by the physical memory effect of MMF, rather than memorizing discrete training samples.

Supplementary References

1 Yariv, A. & Yeh, P. *Photonics: Optical Electronics in Modern Communications* (Oxford University Press, New York, 2007).

2 Li, S. H. et al. Memory effect assisted imaging through multimode optical fibres. *Nature Communications* **12**, 3751 (2021).

3 Plösch ner, M., Tyc, T. & Čižmár, T. Seeing through chaos in multimode fibres. *Nature Photonics* **9**, 529-535 (2015).

4 Redding, B., Popoff, S. M. & Cao, H. All-fiber spectrometer based on speckle pattern reconstruction. *Optics Express* **21**, 6584-6600 (2013).

5 Yuan, S. F. et al. Geometric deep optical sensing. *Science* **379**, eade1220 (2023).
6 Khare, K., Butola, M. & Rajora, S. *Fourier Optics and Computational Imaging* (Springer, 2015).
7 Michalewicz, Z. *Genetic Algorithms + Data Structures = Evolution Programs* (Springer, Berlin, 1996).
8 Lin, X. et al. All-optical machine learning using diffractive deep neural networks. *Science* **361**, 1004-1008 (2018).
9 Ronneberger, O., Fischer, P. & Brox, T. U-net: Convolutional networks for biomedical image segmentation. In *Medical Image Computing and Computer-Assisted Intervention-MICCAI 2015* 234-241 (Springer, Munich, Germany, 2015).
10 Liu, Q. W., He, Z. Y., Tokunaga, T. & Hotate, K. An ultra-high-resolution FBG static-strain sensor for geophysics applications. In *Fourth European Workshop on Optical Fibre Sensors* **7653**, 170-173 (SPIE, 2010).
11 Kuse, N., Ozawa, A. & Kobayashi, Y. Static FBG strain sensor with high resolution and large dynamic range by dual-comb spectroscopy. *Optics Express* **21**, 11141-11149 (2013).
12 Vasu, K. S., Asokan, S. & Sood, A. K. Enhanced strain and temperature sensing by reduced graphene oxide coated etched fiber Bragg gratings. *Optics Letters* **41**, 2604-2607 (2016).
13 Li, B. C. et al. Dilated convolutional neural networks for fiber Bragg grating signal demodulation. *Optics Express* **29**, 7110-7123 (2021).
14 Yamaguchi, T., Kawashima, H., Matsuda, H. & Shinoda, Y. Improvement of multiplexing capability of fiber Bragg gratings using convolutional neural network. In *Optical Fiber Sensors* W4-80 (Optica Publishing Group, 2023).
15 Fujiwara, E., Silva, L. E., Marques, T. H. R. & Cordeiro, C. M. B. Polymer optical fiber specklegram strain sensor with extended dynamic range. *Optical Engineering* **57**, 116107 (2018).
16 Murray, M. J., Davis, A., Kirkendall, C. & Redding, B. Speckle-based strain sensing in multimode fiber. *Optics Express* **27**, 28494-28506 (2019).
17 Wang, X. C., Wang, Y. F., Zhang, K. T., Althoefer, K. & Su, L. Learning to sense three-dimensional shape deformation of a single multimode fiber. *Scientific Reports* **12**, 12684 (2022).
18 Li, G. D. et al. Fiber specklegram torsion sensor based on residual network. *Optical Fiber Technology* **80**, 103446 (2023).
19 Wang, X. et al. An Ultrasensitive Fiber-End Tactile Sensor With Large Sensing Angle Based on Specklegram Analysis. *IEEE Sensors Journal* **23**, 30394-30402 (2023).
20 Lu, S., Tan, Z. W., Ji, W. J. & Zhang, D. N. A spatial domain multiplexing technology for fiber specklegram sensor. *Optical Fiber Technology* **81**, 103505 (2023).
21 Wang, X. et al. A reflective multimode fiber vector bending sensor based on specklegram. *Optics & Laser Technology* **170**, 110235 (2024).
22 Yuan, H. et al. Torsion and bending sensing based on the specklegrams from a coupled few-mode multi-core fiber. *Optics Communications* **569**, 130732 (2024).
23 Momeni, A. et al. Training of physical neural networks. *Nature* **645**, 53-61 (2025).
24 Wright, L. G. et al. Deep physical neural networks trained with backpropagation. *Nature* **601**, 549-555 (2022).
